# The revolutionary developmental biology of Wilhelm His, Sr.

**DOI:** 10.1111/brv.12834

**Published:** 2022-02-01

**Authors:** Michael K. Richardson, Gerhard Keuck

**Affiliations:** ^1^ Institute of Biology, University of Leiden, Sylvius Laboratory Sylviusweg 72 Leiden 2333 BE The Netherlands; ^2^ Geißspitzweg 8 Frankfurt 65929 Germany

**Keywords:** Wilhelm His, mechanobiology, evolution and development, evo‐devo, gastrulation, germ layer, morphogenesis, allometry, Entwicklungsmechanik

## Abstract

Swiss‐born embryologist Wilhelm His, Sr. (1831–1904) was the first scientist to study embryos using paraffin histology, serial sectioning and three‐dimensional modelling. With these techniques, His made many important discoveries in vertebrate embryology and developmental neurobiology, earning him two Nobel Prize nominations. He also developed several theories of mechanical and evolutionary developmental biology. His argued that adult form is determined by the differential growth of developmental primordia. Furthermore, he suggested that changes in the growth parameters of those primordia are responsible for generating new phenotypes during evolution. His developed these theories in his book ‘*Our Bodily Form*’ (*Unsere Körperform)*. Here, we review His's work with special emphasis on its potential importance to the disciplines of evolutionary developmental biology (evo‐devo) and mechanobiology.

## I. INTRODUCTION

With the recent death of Leipzig anatomist Wilhelm His [Sr.], we have seen the burial of one of the most idiosyncratic of research personalities. Certainly not a trail‐blazer like Carl Gegenbaur, or a fighter like his great opponent Ernst Haeckel, but a quiet scholar who worked with real German thoroughness… and immersed himself with real German sensibility in the details of phenomena … nonetheless, none of the other outstanding anatomists of the nineteenth century was treated with such hostility – almost hatred – as Wilhelm His. (Rawitz, 1904, p. 308)

**Fig. 1:**
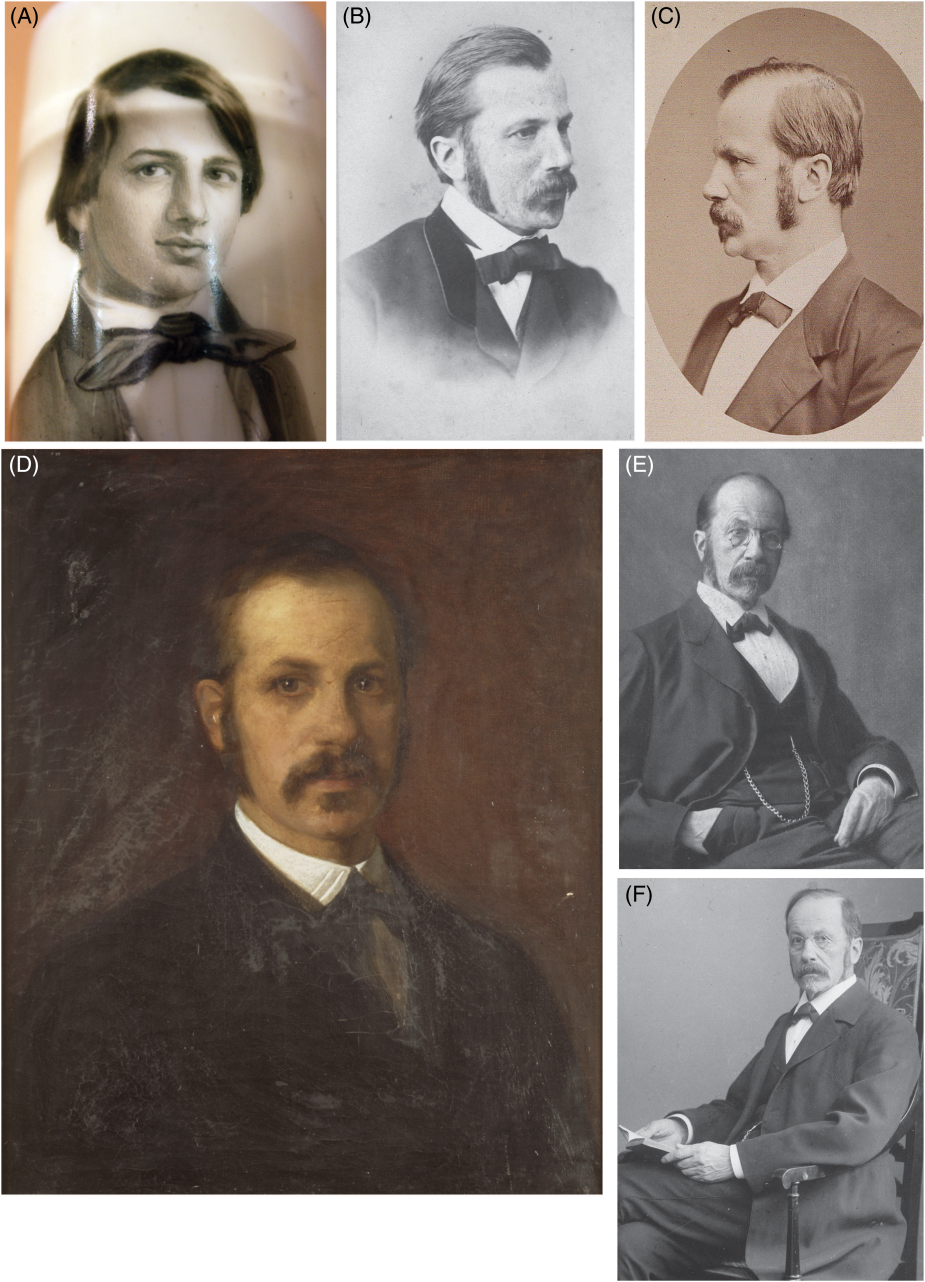
Portraits of Wilhelm His. (A) 1849, portrait, aged 18, on the bowl of a pipe, from a watercolour by Hans Burckhardt; Anatomisches Museum, Basel. Photograph M. K. Richardson. (B) Photograph, 1872; courtesy of Roger and Hugo Kurz. (C) Photograph; courtesy of Fotosammlung 11, Universitätsbibliothek, Leipzig. An identical image in the Alan Mason Chesney Medical Archives is inscribed: ‘G. Brokesch 1882–83 Leipzig’. (D) Painting (oil on canvas), 62 × 49 cm, signed Albert Winther, dated 189?; Kunstbesitz der Universität Leipzig, inventory no. 1951:004, photographed by Karin Kranich; image rights: Kustodie der Universität Leipzig (for more information, see Supplementary Note S1). (E) Undated photograph, courtesy of the History of Medicine Collections, David M. Rubenstein Rare Book and Manuscript Library, Duke University. (F) Photograph, circa 1900 (source as B). B and F are figs 3 and 6 in Kurz (1992) from where the information provided here was taken.

**Fig. 2:**
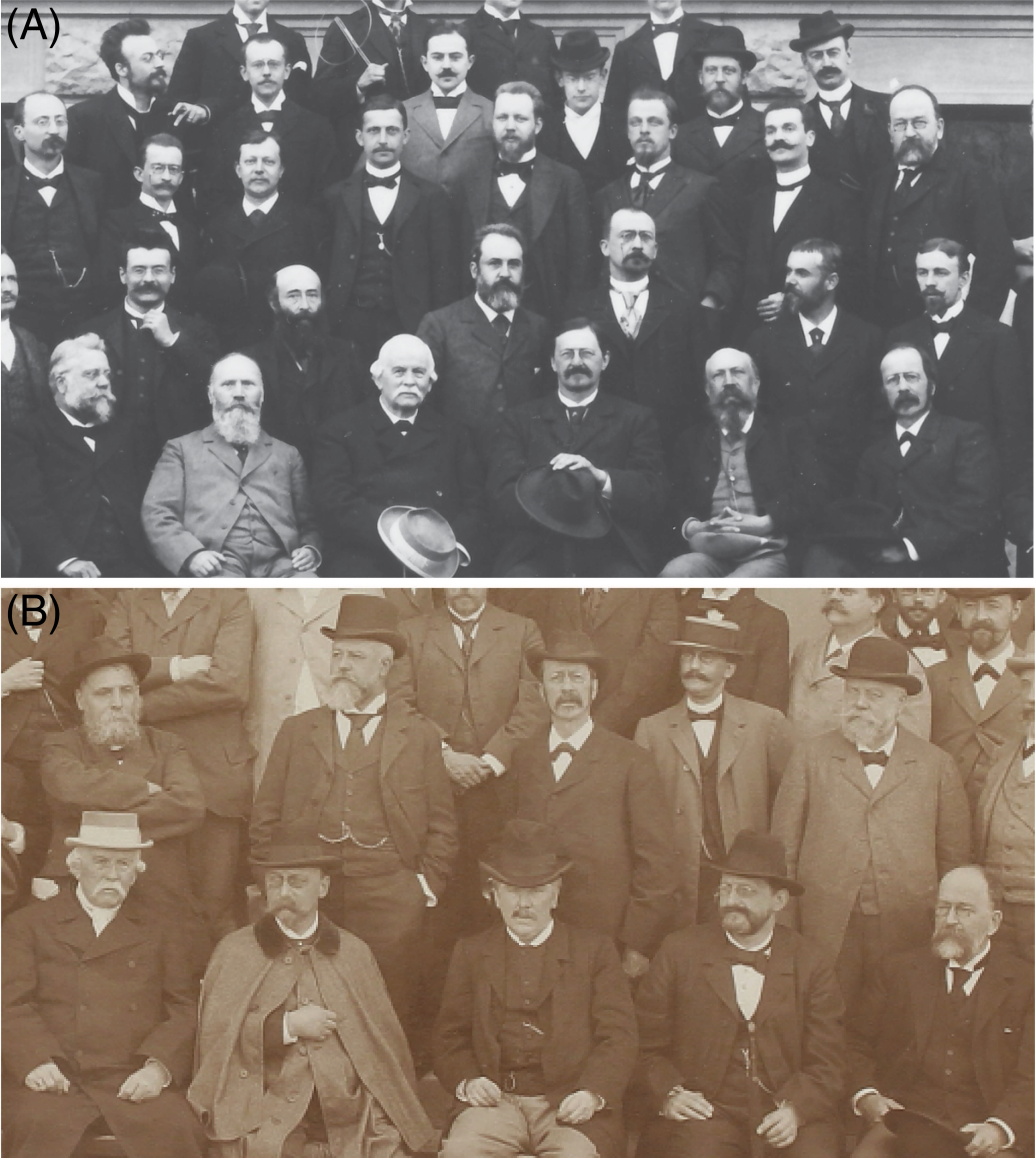
Wilhelm His and other delegates at meetings (1895 and 1897) of the Anatomische Gesellschaft. (A) Basel (17–20 April, 1895), the meeting from which the ‘Basel’ *Nomina Anatomica* took its name (His, 1895b). Wilhelm His: seated, front row, far right. (B) Tübingen, 21–24 May, 1899. Wilhelm His: middle of second row, with round‐framed glasses. Images courtesy the Archive of the Anatomische Gesellschaft. For key to sitters see Fig. S1.

**Fig. 3:**
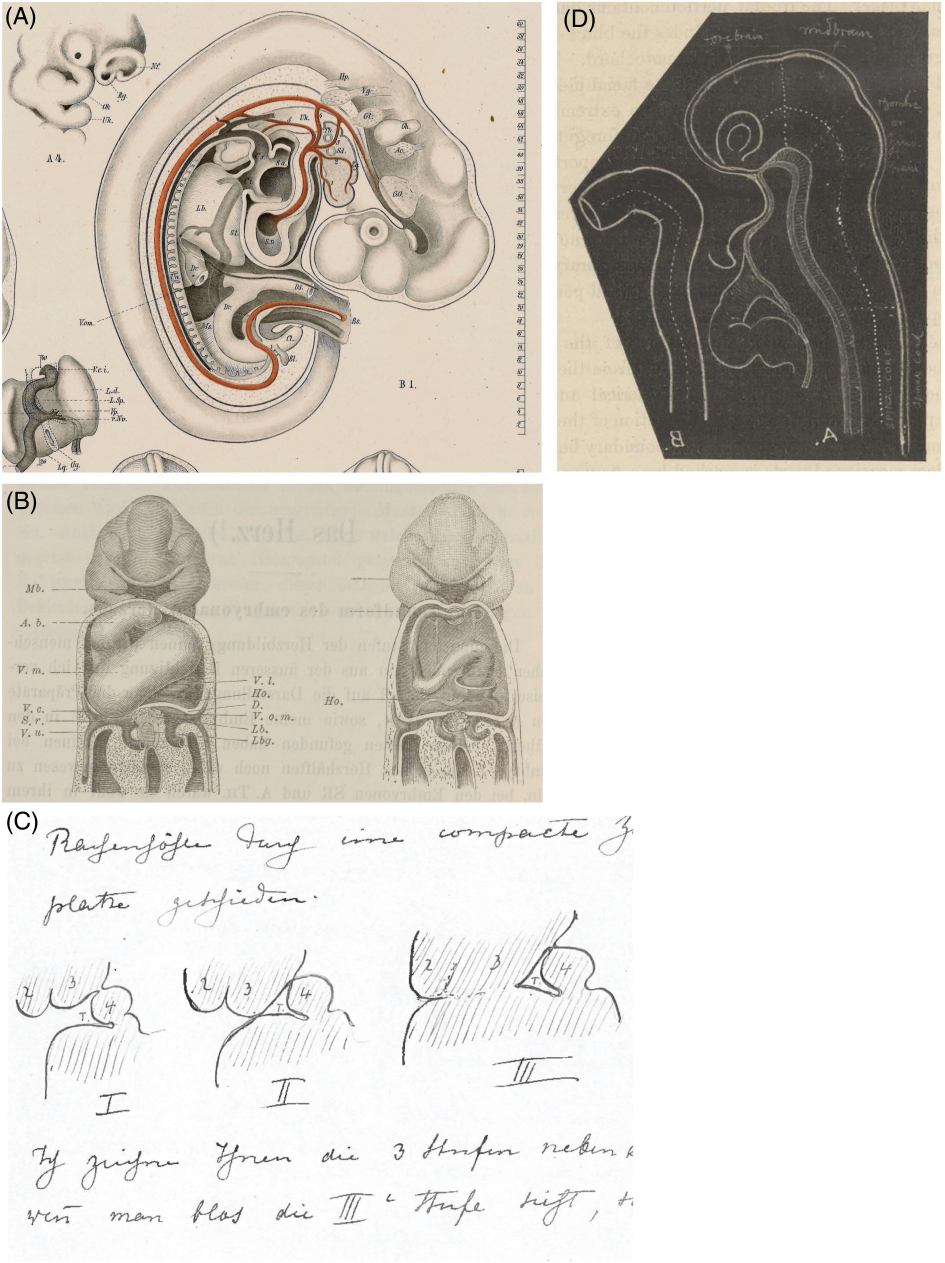
Scientific illustrations by Wilhelm His. (A) Fig. B1 from Plate VII in His (1880). Reconstruction of a human embryo from serial sections. The corresponding section numbers are shown by the scale (right). Note the semi‐transparent rendering of the liver (*Lb*) to reveal the intrahepatic course of the inferior vena cava. A note printed at the bottom of the plate attributes the drawings to His. (B) Figs 78 and 79, p. 130 in His (1885). Note the use of cut‐away layers to expose the endocardial tube. (C) Pen‐sketch by His, in a letter dated 20th March, 1889 to his former student Franklin Mall. His refers to a discussion between them over the developmental origin of the thymus. From the Mall Papers (Wilhelm His), image courtesy of the Alan Mason Chesney Medical Archives of the Johns Hopkins Medical Institutions. (D) Blackboard drawing made by His during his lecture to a meeting of the Anatomical Society of Great Britain and Ireland at the Royal Dublin Society on June 11th, 1897. The drawing was photographed and reproduced as fig. 1 in His (1897a). Image courtesy the Wellcome Collection.

Wilhelm His, Sr. (1831–1904) (Figs 1 and 2; Supplementary Note S1) was a Swiss‐born physician who pursued a career in embryological and anatomical research in Basel and then Leipzig. Landmarks in his career are given as online Supporting Information in Table S1. His published on a wide range of scientific topics (Fig. S2). For example, he showed that the lymphatic system is a closed system (see Table S2 for a glossary of terms); and he produced an early example of forensic craniofacial reconstruction, using the supposed skeletal remains of Johan Sebastian Bach (Supplementary Note S2). He also made important contributions to vertebrate embryology and developmental neurobiology, including neuron theory (Supplementary Note S3). His's success in anatomical research was due in part to his ability to understand the complex three‐dimensional (3‐D) relationships of structures, and how they change during development (Fig. 3).

In recent years, several historians and biologists have discussed His's research (e.g. Gould, 1977; Maienschein, 1991; Nyhart, 1995; Hopwood, 1999, 2000, 2002, 2005, 2007, 2012, 2013; Richardson & Keuck, 2001; Hoßfeld & Olsson, 2003; Brauckmann, 2006; Laubichler, Aird & Maienschein, 2007; Richards, 2008; Dupont, 2017; Glover *et al*., 2018). His is well‐known for his ‘mechanical’ theories of development. He wrote: ‘Embryology and morphology cannot proceed independently of all reference to the general laws of matter, – to the laws of physics and of mechanics’ (His, 1888a, p. 293). Our aims in this review are to explore these mechanical theories; to examine how His applied these theories to evolutionary questions; and to consider the potential relevance of His's theories to the modern disciplines of evolutionary developmental biology (evo‐devo) and mechanobiology.

We start by examining His's early research and show how it formed the basis of theories that he would advocate for most of his career. Then, we consider his (1875) book ‘*Our Bodily Form*’, which contained new hypotheses about vertebrate developmental mechanisms and their relationship to evolution. Finally, we examine how His's theories have been received by scientists and examine them in the light of current scientific knowledge. We should note that His's son, Wilhelm His Jr. was also a physician and scientist; he described the atrioventricular bundle in the heart (Roguin, 2006; Anderson & Mori, 2016).

## II. WILHELM HIS'S EARLY RESEARCH

As a medical student, His spent three semesters at Berlin University. During that time, he and other students were invited by Robert Remak, one of the lecturers, to his home on the Unter den Linden. There, Remak had created a makeshift embryology laboratory, and he showed the students how to prepare chicken embryos for microscopical analysis (His, 1868b, p. 50; His, 1903, p. 26). These interactions with Remak stimulated in His an enduring interest in early chicken development. Remak stressed the importance of understanding the germ layers in relation to histogenesis: the development of mature tissues from precursor cells (Table S2). His took up these issues in a paper entitled ‘The membranes and cavities of the body’ (His, 1865b).

### (1) Membranes and cavities

During the nineteenth century we see three great steps in anatomy: general anatomy, associated with the name of Bichat, the cell doctrine with that of Schwann, and histogenesis with that of His. (Mall, 1905, pp. 144–145).

In his paper ‘Membranes and cavities’, His coined the name ‘endothelia’ for epithelia that arise from the mesoderm, and which come to line body cavities, and the blood and lymphatic vessels (His, 1865b, p. 18). This paper contains highly original speculations on developmental mechanisms. Those speculations appear to be informed by his unpublished histological studies of chick embryos. His noted that embryonic cavities develop from splits in the mesoderm, and suggested that this splitting might be mediated by mechanical forces. When a split develops, he argued, it be becomes filled with fluid. This leads to swelling of the extracellular matrix, and the resulting pressure is modulated by elastic fibres in the matrix. Further, His argued that mechanical forces in the matrix might actually influence cell behaviour:

The remarks above concern an influence of mechanical moments on the development of connective tissue, which we can call *directive* [richtenden]; in this case, an indifferent tissue mass is assumed, and the special way of developing that it experiences under given external influences is followed. Now, mechanical influences, along with everything else that comes under the broad concept of stimuli, can act in a *determinant* [bestimmend] way upon cell proliferation and thereby on the formation of tissues. (His, 1865b, p. 30, his italics; the words in square brackets are those used in the German original)

His also speculated that ectoderm and endoderm might provide a chemical signal to the mesoderm which stimulates growth and blood vessel development (His, 1865b, p. 33).

**Fig. 4:**
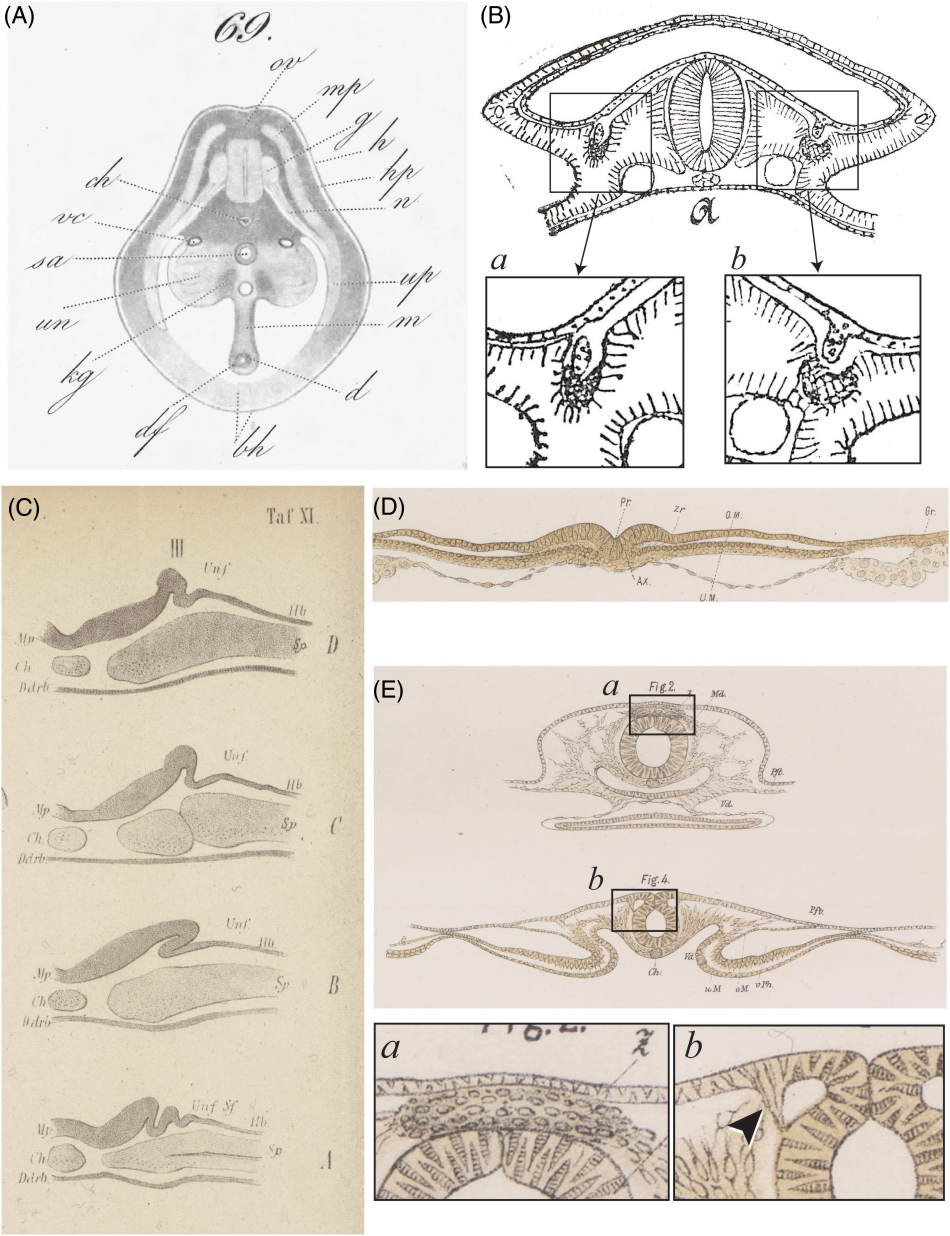
Conflicting hypotheses of the origin of the neural crest and kidney duct. (A) Plate V, fig. 69 in Remak (1855). Transverse slice (possibly from a fresh embryo) through the trunk of a chicken embryo viewed with transmitted light. Individual cells are not shown; the tissue is simply block‐shaded, with selected mesodermal derivatives shown in a paler shade. (B) Fig. IVa, p. 32 in Hensen (1903) showing a transverse section with what Hensen believed to be the precursor of the nephric duct arising from a cell mass in the ectoderm; the mass grows ventrad towards the mesoderm and then becomes detached (our boxes *a* and *b*). (C) Plate XI (III) in His (1865a) *A*–*D* (from bottom to top) are sections from the same chicken embryo showing what His thought was the precursor of the nephric duct (Urnierenfalte, *Unf*.). (D) Plate VI, fig. III (4) in His (1868b). Transvere section through a chicken embryo showing the intermediate groove (*Zr*), the supposed precursor of the peripheral ganglia. (E) Plate VII, box I, figs 2, 4 in (His, 1868b). Our box *a* shows detail of intermediate cord (*Z*); our box *b* shows precursors of the ganglia as an inverted pyramid of tissue (arrowhead). Note that His's figures show individual cells, a reflection of his improved histological techniques.

His challenged Remak's view that the peripheral nervous system develops from the mesoderm, arguing (in agreement with data available today) that it develops from the ectoderm (Fig. 4; Table S3; Remak, 1855, p. 44; His, 1865b; pp. 7–9). His also disputed Remak's view that the nephric duct arises from the mesoderm, arguing for an ectodermal origin (the data available today are consistent with Remak's view). His later changed his mind when he realised that what he had taken to be the nephric precursor was actually the primordium of the peripheral nervous system. With this primordium, His had discovered the neural crest (a term later coined by Marshall; Fig. 4; Tables S2 and S3). His (1865b, p. 8) suggested that such disagreements about the origins of tissues arose because of the limited resolution of the histological techniques of the time.

### (2) Improved histological techniques

Like my predecessors, I had little success in achieving a uniform view of the genesis of the nervous system by way of observation, and I could reach only indirect conclusions about the developmental history of ganglia. The obstacle to decisive observations seemed to lie in deficient technology, and this has kept me working to improve the technique of making sections as fine as possible. (His, 1868b, p. v)

A lack of adequate histological techniques was recognised as an obstacle to progress in embryology (Kölliker, 1876, pp. 102–103; Bischoff, 1877, pp. 2–6; Balfour, 1881, pp. 124–125; Hertwig, 1906, p. 2). The main difficulty lay in obtaining sufficiently thin sections. For most purposes, histologists cut tissue slices by hand with a razor (e.g. His, 1856, p. 3), but young embryos are too delicate to be sectioned in this way. Instead, they were dissected, while living, in a dish of water or saline (Remak, 1855, p. xxxvi; Dursy, 1866, pp. 11–14), essentially applying the dissection techniques of gross anatomy to embryos (Hertwig, 1906, p. 2). With such crude techniques it was impossible to resolve the details of early development, or to adequately employ the 3‐D reconstruction techniques that His had invented (His, 1887, pp. 383–384; Hopwood, 1999, 2000, 2002, 2012); those techniques required an unbroken series of sections of consistent thickness.

His addressed these problems by developing a protocol for the infiltration‐embedding of embryos with paraffin wax (van der Lem *et al*., 2021). Edwin Klebs had introduced paraffin wax to histology (Klebs, 1867) but soon abandoned it (Klebs, 1869, pp. 164–165) because wax does not penetrate watery tissue. His overcame this problem by dehydrating the embryos in alcohol and clearing them in oil of lavender, which is miscible with both alcohol and molten wax, and therefore acts as an intermediate reagent.

**Fig. 5:**
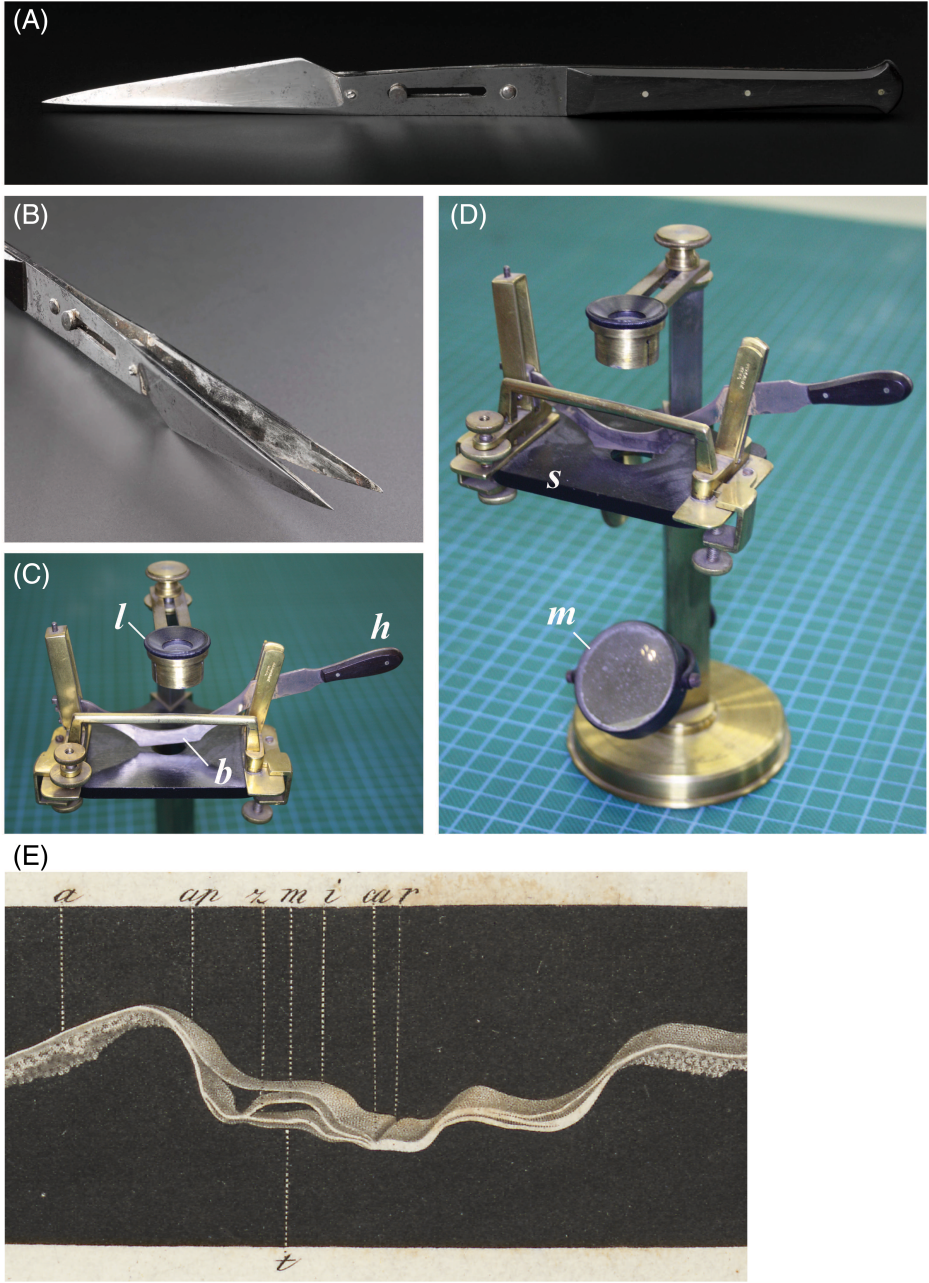
Early microtechniques before the introduction of His's microtome. (A, B) Valentin double‐knife made by Weiss, London, *c*. 1860. Photograph: Science and Society Picture Library, Science Museum, London (object number A135073), Wellcome Trust loan. (B) Detail of blades. Oschatz (1844, p. 129) stated that Valentin used a design of Purkyně's, but Valentin denied this (Valentin, 1845, p. 55 n.). (C, D) Hensen's ‘section‐cutter’; Key: *b*, blade; *h*, handle of chopper; *l*, lens; *m*, sub‐stage mirror; *s*, stage. The device was made by the instrument maker Beckmann (Hensen, 1863, p. 82 n.1). Anatomisches Museum, Basel; Photograph: M. K. Richardson. (E) Thick tissue slice from a chicken embryo, possibly cut underwater from a fresh embryo. From fig. 1 on p. 5 of Reichert (1861), cropped. Image courtesy of the University of Leiden, Special Collections Library.

**Fig. 6:**
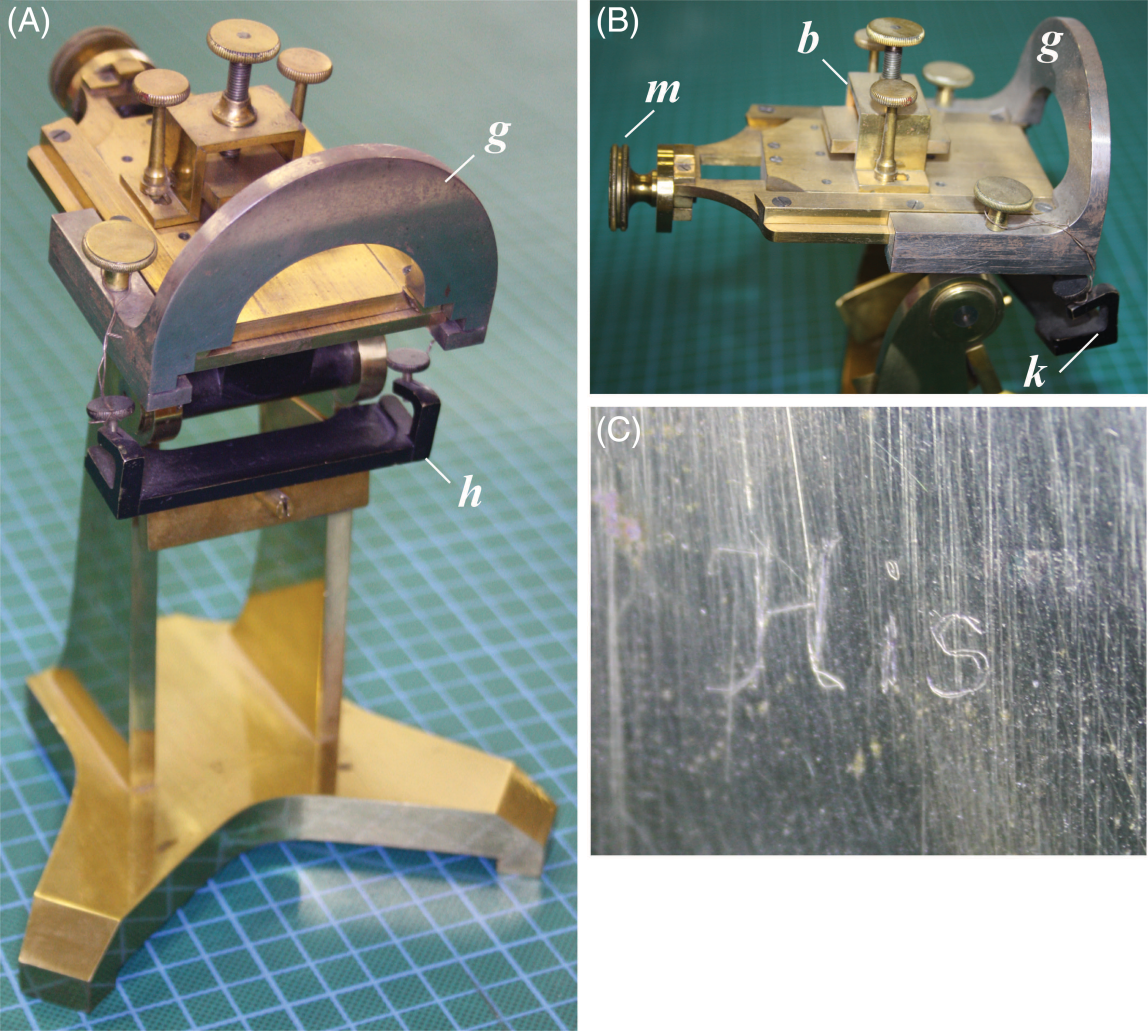
The microtome of Wilhelm His. (A) Front view; (B) side view of stage; (C) engraved name ‘His’ on the strut. Background gridline spacing: 1 cm. The blade, held in the blade‐holder *h* (fastened with wire for display purposes) is drawn downwards, the holder steadied against the flat face of *g*. Between cuts, the block clamped in *b* is advanced under the arch of *g* using the micrometer screw (*m*). Anatomisches Museum, Basel; photograph: M. K. Richardson.

The sectioning of paraffin‐embedded embryos by hand was very time consuming (His, 1870a, p. 229). There were several mechanical aids to sectioning then available such as Valentin's ‘double‐knife’ (Fig. 5A, B), and Hensen's ‘section‐cutter’ (Fig. 5C, D; Hensen, 1863, p. 82; His, 1887, p. 383). His designed his own microtome (Fig. 6) for the free‐hand sectioning of paraffin‐embedded embryos. This microtome allowed sections to be cut individually (rather than in a ribbon; His, 1868b, p. 181) at His's preferred section thickness of 50 μm (His, 1887, p. 383). His's new workflow of infiltration embedding, sectioning and 3‐D modelling formed the basis of His's studies on the early embryology of vertebrates.

### (3) Studies of early chicken development

In the 1860s and early 1870s, His published research on the early embryology of sharks, teleosts and the chicken (see bibliography in Fick, 1904). Among these works was a monograph on the early development of the chicken (His, 1868b) that established His's name as an embryologist (Marchand, 1905, pp. 329–330).

#### (a) The growth law and organ‐forming regions

The entire development of the organism can be derived from a growth law which originates as a relatively simple function of space and time. (His, 1868b, p. 220)

His's chicken embryology monograph (His, 1868b) was partly descriptive, partly theoretical. The theoretical part was a model of morphogenesis based on the differential growth of embryonic tissues. He wrote: ‘The first step in any attempt to deduce a physical form must therefore be the search for [a] basic law of growth’ (His, 1868b, p. 184). The values of the embryonic growth parameters, he says, are unique to each species and change during evolution. In our view, His's growth law was the first comprehensive model of morphogenesis and morphological evolution with a testable mechanism.

His measured the dimensions of chicken embryos at different stages of development. Using tissue thickness as a proxy for growth rate, he found gradients of growth rate in the blastoderm along all three anatomical axes (His, 1868b, pp. 188–190). His mapped the gradients onto the embryo using a system of 3‐D Cartesian coordinates (His, 1867a, pp. 620–621).

The causes of tissue growth, according to His, are cell proliferation, increase in cell size, cell movements where cells slide past one another (His, 1867a, p. 618; His, 1868b, p. 53), and changes in cell shape (His, 1868b, p. 53). The resultant increase in the volume of the tissues creates mechanical pressures which deform the tissues in accordance with their inherent elasticity (His, 1868b, pp. 52–53). His further argued that differential growth causes some regions of the blastoderm to become thicker — and therefore less elastic — than others. As a result, tissue elasticity becomes unequally distributed. Ultimately, the pressure of differential growth on an unevenly elastic embryo creates tensile and compressive forces that cause the blastoderm to fold.

His used the growth law to explain phenomena such as gastrulation and other morphogenetic movements (His, 1867a, p. 621). It also provided a possible mechanism for regional specification. His argued that the folding of the blastoderm divides it into territories with different developmental fates (His, 1868b, p. 197). These territories or ‘organ‐forming embryonic regions’ (His, 1875, p. 19) are similar to von Baer's ‘primitive organs’ (epithelial tubes, etc.; von Baer, 1837, p. 65), except that they are completely undifferentiated.

#### (b) The modelling of mechanical forces in development

**Fig. 7:**
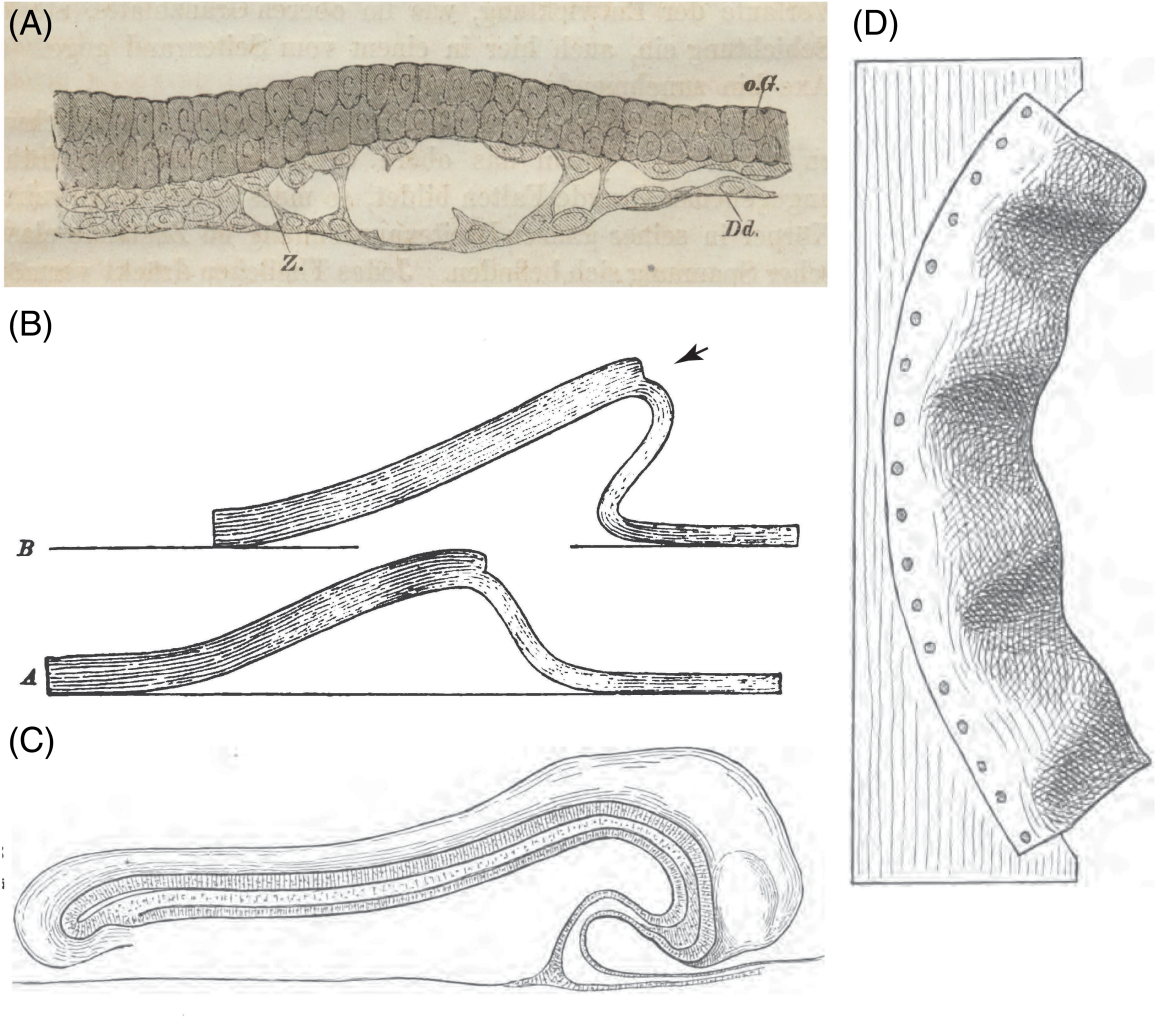
Tensile and compressive forces in development. (A) Fig. 44 in His (1875) showing the formation of a split in the ventral germ layer (*Dd*) of a bilaminar chicken embryo, resulting from the lateral tension produced by the faster growth of the upper layer (pp. 58–59). The loose cells torn from the lower layer will become the lower leaf of the mesoderm. *oG*, epiblast; *Z*, cell stretched across the gap. (B) Fig. 10 in His (1894). Sheet of clay with a sudden transition (arrow) from thick (8 mm) to thin (4 mm). In the lower illustration there is a span of 15 cm, but in the upper illustration the sheet has been pushed from the left to decrease the span to 12 cm. The result is an asymmetric fold resembling the head fold in C. (C) Fig. 26 in His (1894), ray (*Torpedo* sp.) embryo showing the head fold. Original rotated 90° anticlockwise and horizontally reflected by us. (D) Fig. 17 in His (1894; also reproduced as fig. 3 in Hopwood, 1999). A strip of leather, forced into a curve and fixed along one side. The resultant forces throw the leather into folds resembling intersomitic clefts.

His used clay, metal, rubber and other materials to model development (Table S4; His, 1867a, p. 623; His, 1868b, p. 182; His, 1894; Hopwood, 1999, 2002). He was also interested in the geological literature on tangential pressures formed in the Earth's crust as it cooled, and how these might cause the land to rise up as mountain ranges. His thought, by analogy, that pressure established by differential growth in the embryo might cause the folding and splitting of tissue layers [Fig. 7; His, 1894; Hopwood, 1999, p. 470 note (n.) 16]. A similar analogy was later drawn by (Roux, 1895a, pp. 6–7).

In addition to modelling with physical materials, His asked Eduard Hagenbach to model development mathematically. A theoretical, circular elastic plate, representing the blastodisc, was transformed by specified growth parameters (His, 1868b, pp. 191–194). According to Hagenbach's calculations, the circular plate would be transformed into an ellipse (Brauckmann, 2006, p. 401; Hopwood, 2015, pp. 97–98).

### (4) The growth law in conflict with the biogenetic law

His did not incorporate Darwinian theory into his models of development and evolution. He appreciated the explanatory potential of Darwin's ideas, but felt that they were unproven (His, 1868b, pp. 223–224). Ultimately, His thought that Darwinian theory was irrelevant: all that was needed to explain morphological evolution were the mechanics of tissue growth, and species‐differences in the growth law (His, 1868b, pp. 211–213). His's agnosticism towards Darwinian theory is in contrast with the pro‐Darwinian stance of Ernst Haeckel.

Haeckel shared His's desire to explain development on ‘physiological’ or physico‐chemical grounds [Haeckel, 1866, volume (vol.) 1, p. 107; Haeckel, 1866, vol. 2, p. 295]. But while His believed that the embryo contains all causal explanations for its own development, Haeckel believed that the causal mechanisms of development – adaptation and heredity – lay outside the egg, in its phylogenetic history (Haeckel, 1866, pp. 50–60; Richards, 2008, pp. 282–286; Abzhanov, 2013; Olsson, Levit & Hossfeld, 2017).

**Fig. 8:**
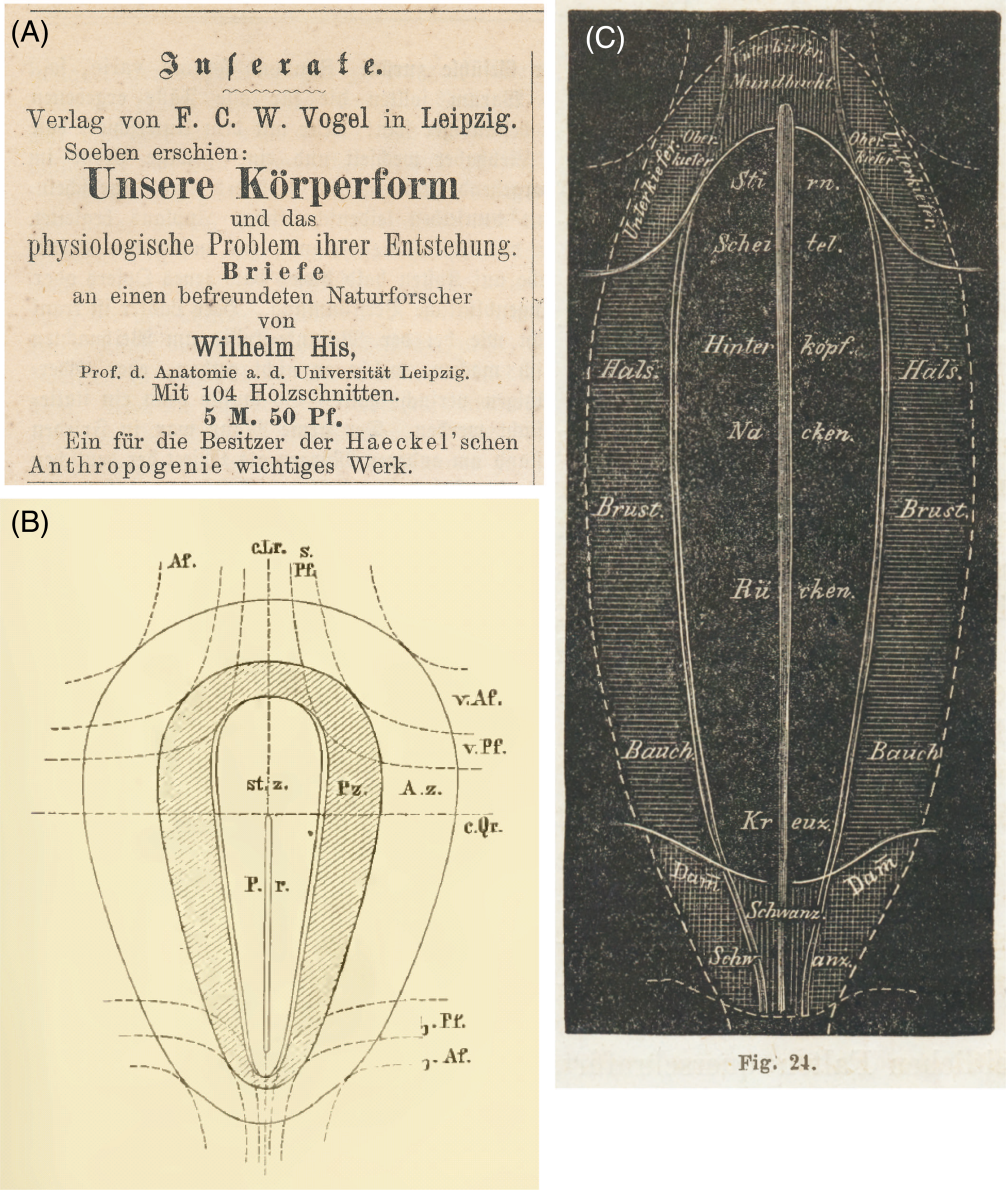
(A) Advertisement for ‘*Our Bodily Form*’ in *Gegenwart*, Issue 13, March 27th, 1875, p. 208. (B) Figure on p. 45 of His (1868b) showing lines of folding that subdivide the embryo into organ‐forming regions. (C) Fig. 24 from His (1875). Fate map showing organ‐forming regions demarcated by folding of the embryo. Key: *Bauch*, abdomen; *Brust*, ventral thorax; *Damm*, perineum; *Hals*, ventral region of neck; *Hinterkopf*, occipital region; *Kreuz*, sacral spine; *Mundbucht*, stomodaeum; *Nacken*, cervical spine; *Oberkiefer*, maxilla; *Rücken*, thoracic spine; *Scheitel*, vertex; *Schwanz*, coccygeal region; *Stirn*, forehead; *Unterkiefer*, mandible.

His and Haeckel's scientific differences quickly became acrimonious (Supplementary Note S4; Spitzer, 1886; Rádl, 1909; Maienschein, 1991; Nyhart, 1995, pp. 188–190; Richards, 2008, pp. 280–291; Hopwood, 2015, pp. 123–126; see Supplementary Note S5 for His's clashes with other scientists). It is likely that his angry exchanges with Haeckel prompted His to write his book, ‘*Our Bodily Form*’, as a riposte (Richards, 2008, p. 298). Indeed, one advertisement for ‘*Our Bodily Form*’ specifically recommends it to owners of Haeckel's ‘*Anthropogeny*’ (Fig. 8A). Furthermore, His wrote his book in just a few weeks (His, 1931, p. 25), suggesting perhaps that he was anxious to give a quick reply to Haeckel's most recent attacks (Supplementary Note S4). ‘*Our Bodily Form*’ contains scientific arguments against Haeckel's phylogenetic approach, and personal attacks on Haeckel's scientific credibility. It also contains important new ideas about development and evolution.

## III. *‘OUR BODILY FORM’* (*UNSERE KÖRPERFORM*)

Finally, prompted by my ‘Natural History of Creation’ and ‘Anthropogeny’, His gives a general exposition of the same in his 1875 publication ‘Our Bodily Form’, the most important part of which is particularly directed against me.” (Haeckel, 1875, p. 13)

His's semi‐popular book ‘*Our Bodily Form*’ (His, 1875; often mis‐cited as 1874; see Table S5) is of particular interest because of its many new theories about evolution and development. Its full title can be translated as: ‘*Our Bodily Form and the Physiological Problem of its Origin: Letters to a Natural Scientist Friend*’. A translation of the Table of Contents is provided in Table S5. For other discussions of ‘*Our Bodily Form*’ see Maienschein (1978, pp. 132–134), Maienschein (1981, pp. 95–96), Gould (1997), Hopwood (2015), Picken (1956) and Richards (2008). The ‘natural scientist friend’ alluded to in the title is His's nephew Friedrich Miescher (His, 1897b, p. 18), who incidentally discovered DNA (in pus that he collected from used surgical dressings; Miescher, 1871, pp. 9, 441; His, 1897b; Dahm, 2005).

One of His's stated goals in publishing ‘*Our Bodily Form*’ was to transform embryology into a new discipline of ‘physiological embryology’: a quantitative science with a reductionist, mechanistic approach (His, 1903, p. 35; Mall, 1905, p. 142). He was inspired by Carl Ludwig's transformation of physiology into a ‘new physiology’ that aimed to explain biological phenome in terms of the underlying chemistry and physics, and to do so using the research methodology of the exact sciences: rigorously planned experiments amenable to mathematical analysis (Ludwig, 1852, 1856; His, 1895c; His, 1898; Rádl, 1909, pp. 549–554; Drischel, 1965; Frank & Weiss, 1966; Rosen, 1973; Fye, 1986; Zimmer, 1996).

### (1) Folding and elasticity of the chicken embryo

According to His, the early chicken embryo becomes demarcated by longitudinal and transverse folds in predictable locations (Fig. 8B, C). One of these folds is the Wolffian ridge, named by His in honour of its discoverer Caspar Wolff (His, 1868b, p. 88; Stephens, 1982). His argued that limb buds arise at the points where the Wolffian ridge intersects a transverse fold. To help his reader understand these folds, His suggested that they try folding a piece of paper into the form of a postal envelope, each corner representing the site of a limb bud (Table S4).

The folding is influenced by the elasticity that, according to His, is an intrinsic property of the chicken germinal disc (and the teleost germinal vesicle; His, 1873b, p. 36). His's evidence for that elasticity is the histological appearance of an elastic extracellular matrix in the embryo (His, 1865b, p. 33). In his ‘*Monograph on Calcareous Sponges*’, Haeckel (1872, p. 472 n.) denied this elasticity: ‘the germinal disc … is not elastic!’ and criticised His for his mechanical approach to evolution and development. In ‘*Our Bodily Form*’, His responded by noting that Haeckel provided no evidence for his denial of elasticity, and suggested resolving the issue by taking a germinal disc from an incubated chicken egg and placing it in a dish of serum. The disc, when prodded with a probe, will show its elasticity by springing back into its original shape (His, 1875, pp. 48–49). In any case, Haeckel considered his own phylogenetic approach (as embodied in the biogenetic law), and His's mechanical approach, to be irreconcilable.

### (2) Organ‐forming regions and a fate map

His illustrated a series of ‘organ‐forming regions’ using what we might now call a fate map (Fig. 8B, C). Today, fate maps are constructed using cell marking and other techniques to track the behaviour of cells and their progeny during development (Le Douarin & Kalcheim, 1999). By contrast, His's fate map is a thought‐experiment in which the definitive body regions are projected, hypothetically, onto the early embryo. His suggested imagining the body of a bird or mammal opened along the ventral midline and flattened out:

If you have carried out in your mind this flattening of the body, it will be clear that on the one hand every point on the embryonic region of the blastoderm must correspond to a later organ, or part of an organ, and that on the other hand any organ that develops from the blastoderm must have a preformed primordium in some spatially defined region of the blastoderm. (His, 1875, p. 19)

His later published a theoretical fate map of the neural primordium (fig. 8 in His, 1893a).

### (3) Differential growth in morphogenesis and evolution

His presented measurements from the embryos of various vertebrate species (His, 1875, pp. 208–210) showing that the early embryo is similar in dimensions in the species examined. But, as the embryos become folded into organ‐forming regions, different quantities of tissue are allocated to each region, and the quantity allocated varies among species. His argued that each primordium has its own ‘partial growth law’, and so some regions grow faster than others (His, 1875, pp. 82–83, 152). The brain primordium, for example, has more tissue allocated to it than other regions and grows faster.

The configuration which the organ finally assumes is therefore dependent on the law of its own growth, on its spatial relations with neighbouring parts, and on the growth of the latter. The principle of unequal growth, according to what has been said, also retains its significance as a form‐determining [morphogenetic] principle in the further course of development. (His, 1875, p. 83)

As an example, he modelled the role of unequal growth in the formation of intersomitic clefts using a strip of leather fixed along one side. When deformed into a curve, the leather assumes a series of segment‐like folds (Fig. 7D; His, 1875, pp. 64–65).

### (4) Germ layers and the parablast theory

In 1875, the germ layers were a battleground for diverse opinions (His, 1875, p. 38; Hertwig & Hertwig, 1881, p. 117), mainly because the available histological techniques made it difficult to resolve the germ layers in detail. His's improved histological techniques led him to a new model of germ layer development: the parablast theory (His, 1866a,b, 1868b; His, 1875, pp. 41–44). This theory envisaged a dual genetic origin for the embryo, with the mesoderm largely arising from maternal ‘parablast’ cells that migrate from the ovary into the embryo *via* the white yolk (the pale‐coloured yolk surrounding the embryo). The parablast theory has some similarities with Reichert's idea that the embryo develops from yolk cells (the ‘formative yolk’; Reichert, 1840, pp. 88–96; Reichert, 1843, pp. 23–27; Stricker, 1872, p. 1201).

### (5) Concrescence theory

Thus the primitive rudiment of the body is a flat ring, whose width and thickness is maximal at the future head end, and minimal at the opposite end, the tail. The two lateral halves of the ring come successively into apposition and unite as symmetrical body halves. (His, 1875, p. 198)

**Fig. 9:**
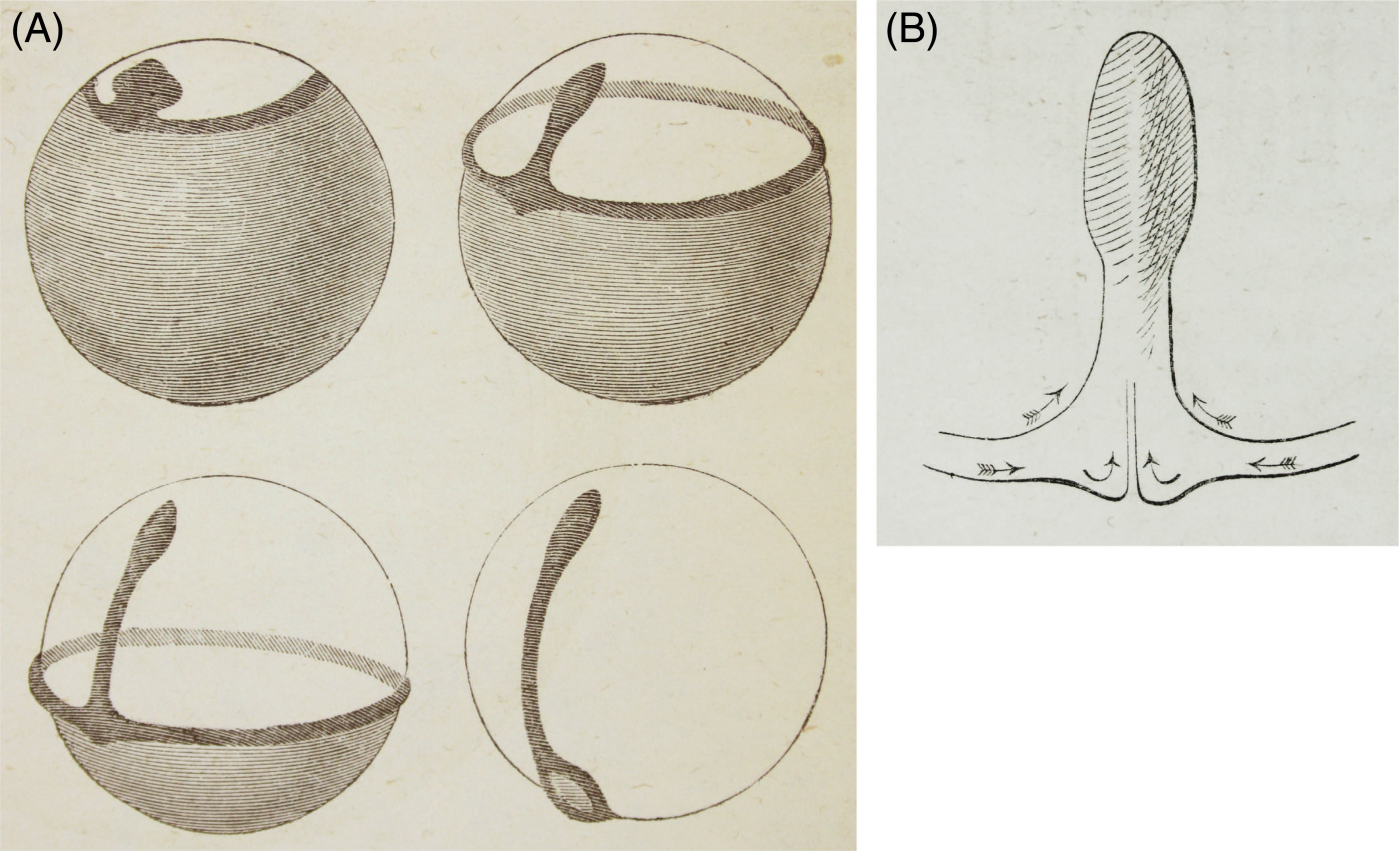
Concrescence in salmon and cat shark embryos. (A) Figs 127–130 in His (1875). This sophisticated schematic uses semi‐transparent rendering to show the marginal ring progressing over the yolk mass during epiboly. The sequence of stages is: top left, top right, bottom left and bottom right. (B) Fig. 126 in His (1875). Schematic illustration of concrescence in bony fish development. Cells from the inner curvature of the germ ring (upper two arrows) contribute to lateral parts of the body; cells from the outer curvature (lower four arrows) contribute to structures closer to the midline. According to the concrescence model, the trunk region is formed by fusion of right and left halves, while the head and tail each consist of one mass from the outset.

Another of His's new ideas – concrescence – was inspired by his studies of teleost development (His, 1874; His, 1875, pp. 188–189; His, 1877b). His suggested that the trunk was formed from left and right embryonic halves that fuse in the midline (Fig. 9). This process of fusion supposedly takes place during epiboly, and became known as ‘concrescence’ (Fig. 9; reviewed by Kopsch, 1904). In one thought experiment, His imagined concrescence as if a ring of rubber tubing were pushed inwards at one point; the resulting two‐layered indentation represents the nascent embryo and the converging halves of the germ ring (His, 1877b, p. 109, n.). A similar idea was perhaps touched on by Lereboullet when he described malformed pike (*Esox* sp.) embryos with a partial split along the longitudinal axis (Lereboullet, 1863; Morgan, 1895, p. 419; Kopsch, 1904, p. 6); it was possible that the split represented the fusion line of two halves.

### (6) Quantitative comparative embryology and a developmental hourglass

#### (a) His's ‘physiognomy’ of embryos

An identity in the external form of animal embryos, as so often asserted, does not exist. Even at an early stage of development, the embryos have their class and their order characters, just as we can scarcely doubt their species and gender, even their individual characters. (His, 1875, p. 201)

**Fig. 10:**
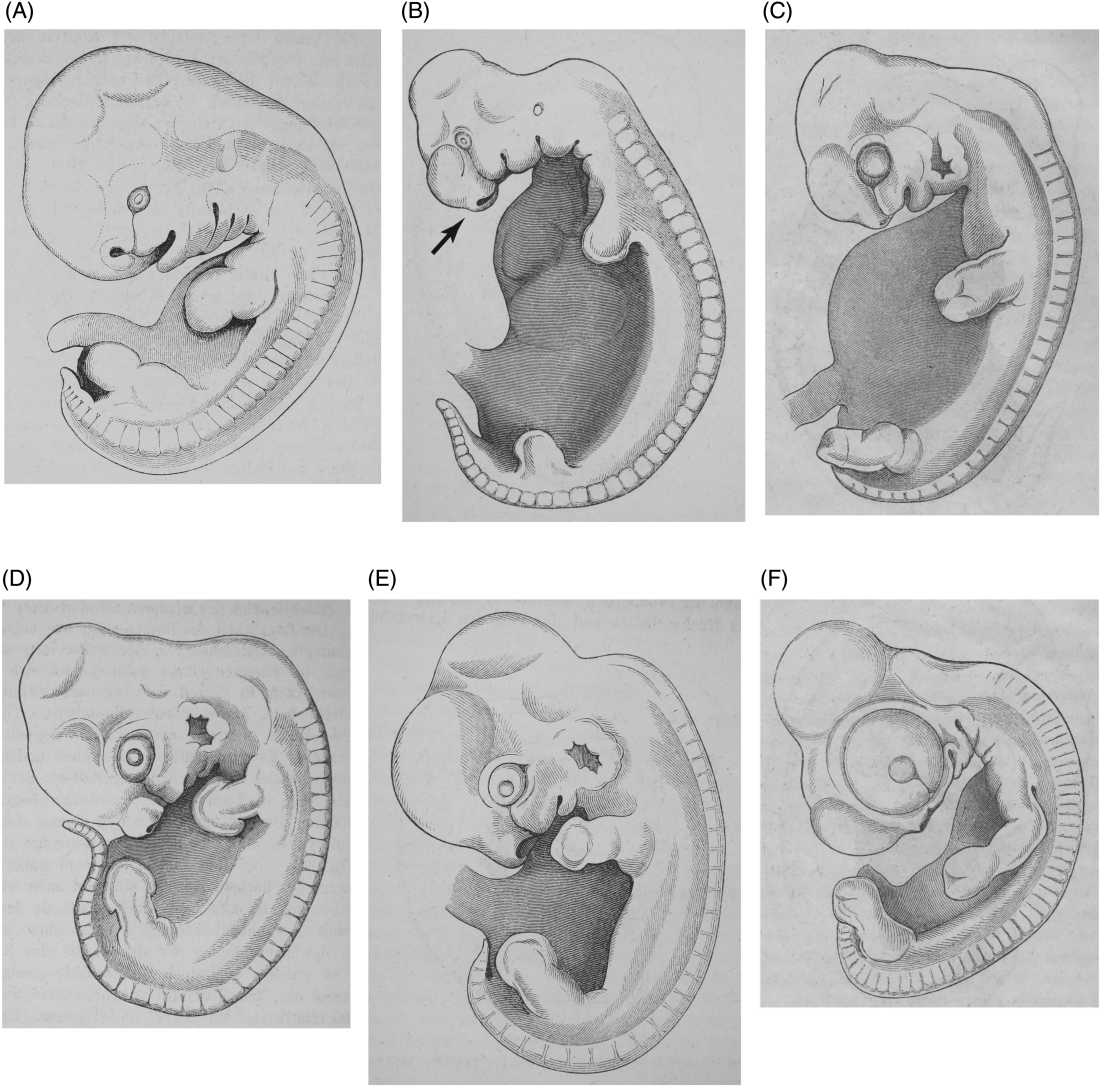
Embryos of six amniotes. From His (1875), limb‐bud stages, left lateral aspect. See a similar composite in Hopwood (2015; fig. 7.14); as Hopwood notes, the embryos were originally on different pages. (A) Fig. 132, human; (B) Fig. 133, pig, arrow indicates the nascent snout described by His; (C) Fig. 134, deer; (D) Fig. 135, rabbit, 14 days (d) post‐fertilisation; (E) Fig. 136, guinea‐pig; (F) Fig. 137, chicken, 5 d post‐fertilisation.

**Fig. 11:**
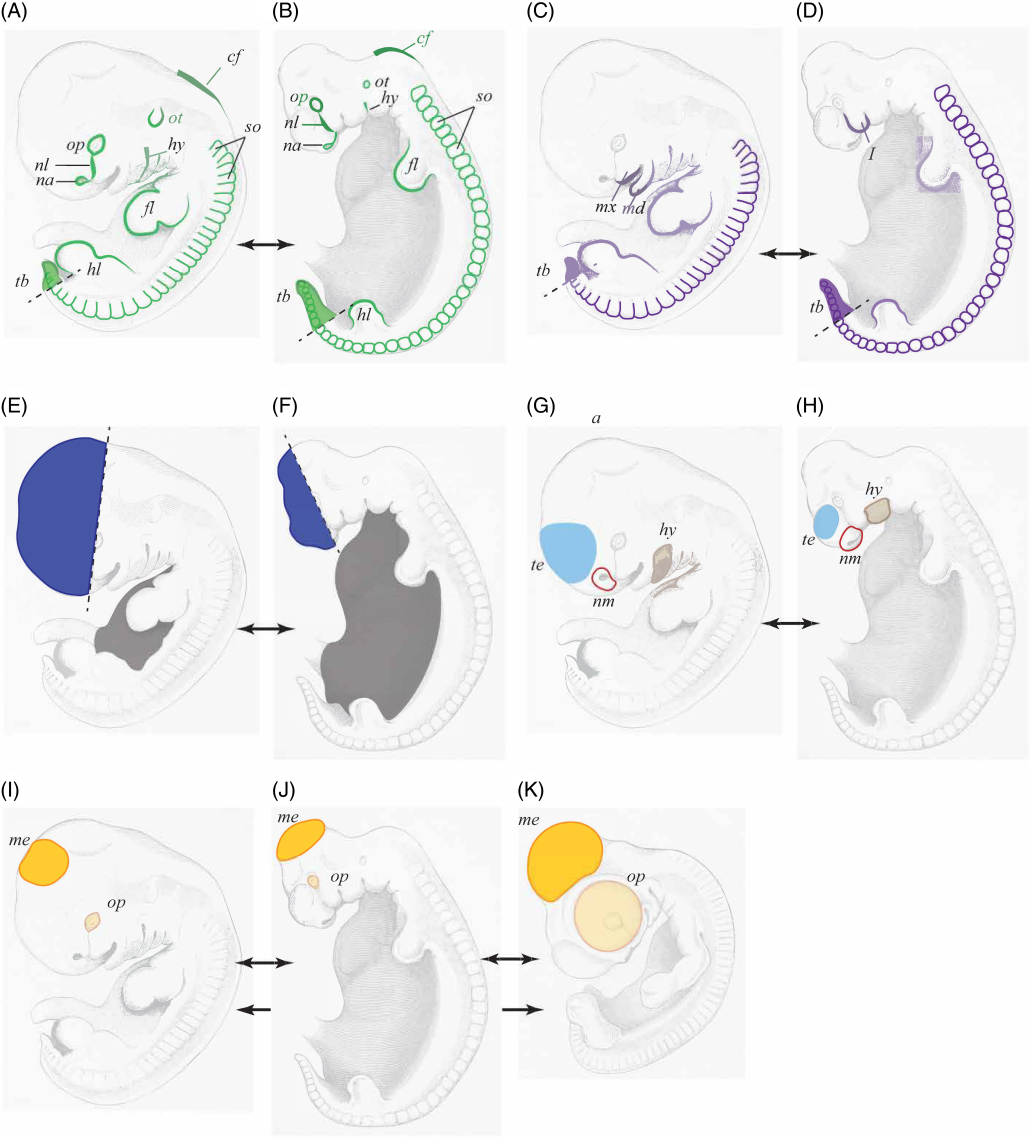
Schematic analysis of some of the six amniote embryos shown in Fig. 10, showing our interpretation of His's (1875, pp. 192–206) text. Double arrowheads indicate the images to be compared in each example. (A, B) Pairwise comparison illustrating that human (A) and pig (B) embryos share many common characters (highlighted in green): (*cf*, cervical flexure; *fl*, forelimb; *hl*, hindlimb; *hy*, hyoid hyomandibular groove; *na*, nasal pit; *nl*, nasolachrymal groove; *op*, optic Anlage; *ot*, otocyst; *so*, somites; *tb*, tailbud. (C, D) Comparison of human (C) and pig (D) showing that common characters present different character states (highlighted in purple). Thus the simple first pharangeal arch (*I*) in D is divided into mandibular (*md*) and maxillary (*mx*) processes in C. His (1875, p. 196) suggested that the modest development of the human tailbud (*tb*) is a foreshadowing of the vestigial state of the adult coccyx. (E, F) Comparison of human (E) and pig (F) embryos showing relatively massive development of the head, rostral to the eye (shaded blue) in the human; and the relatively massive size of the trunk overlying the pleuro‐peritoneal cavity in the pig (grey). (G, H) The human embryo (G) has a larger telencephalon (*te*, shaded blue) than the pig (H). The nasal processes (red outline) give the nasal margin (*nm*) the appearance of a rudimentary snout. The hyoid arch (*hy*, brown) of the pig embryo is ‘plumper’ (His, 1875, p. 195) than that of the human. (I, J, K) The eye (*op*) and midbrain (*me*) together occupy two‐thirds of the area of the head in the chick (K) but less in the human (I) and pig (J). Yellow shading in the two mammals is mainly the lens, whereas in the chicken a large part of the optic cup in addition to the lens is also exposed and shaded. The optic Anlage is much larger in the chicken than in the mammals.

**Fig. 12:**
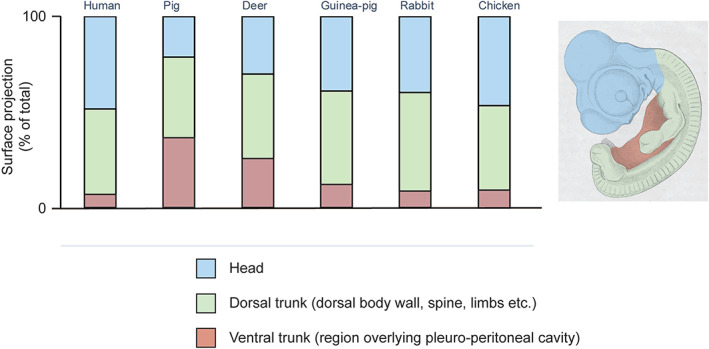
Chart summarising His's morphometric analysis of six amniote embryos. Measurements taken from table in His (1875, p. 201). His notes that the chicken and human embryos have relatively large heads (blue), while the deer and pig both have relatively large pleuroperitoneal regions (pink). The dorsal trunk and limbs combined (green) are similar in relative size in all six species.

**Fig. 13:**
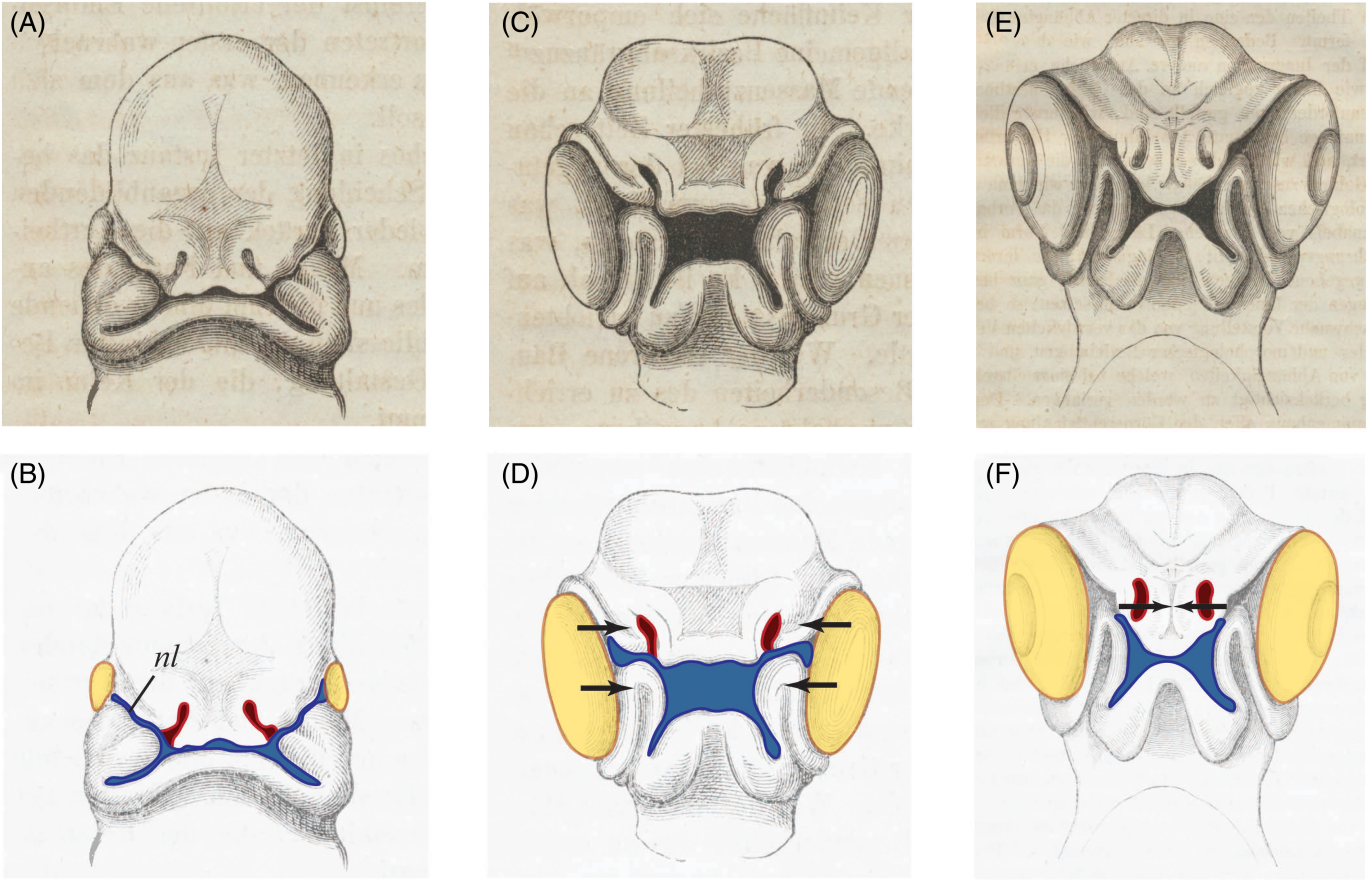
Allometric growth and the development of the beak. Our interpretation of figures of the rostral aspect of the face and text in His (1875, pp. 204–206). (A, B) Fig. 139, 14 day (d) rabbit; (C, D) Fig. 139, 5 d chicken; (E, F) Fig. 140, 6 d chicken. According to His, the eye (yellow) of the chicken embryo grows enormously in size compared with that of the rabbit. As a consequence, the stomodeum (blue) and internasal region in the chicken are laterally compressed (arrows in D), causing the nasal pits (red) to converge (arrows in F). His argued that these changes contribute to the formation of a narrow, protruding beak. Key: *nl*, nasolachrymal duct.

As part of his attack on the biogenetic law, His tried to disprove Haeckel's claim that embryos of different vertebrate species are identical in morphology during organogenetic stages (reviewed by Richardson & Keuck, 2002). His compared six amniote embryos at limb‐bud stages (Figs 10, 11, 12) and quantified their differences using a simple morphometric analysis: he traced the outline of each embryo onto paper, cut round the outlines, then weighed the cut‐outs to estimate their surface area. His analysis showed, for example, that the head of the human embryo is massive, and its body small, compared to the pig embryo. His noted the massive development of the eye in the chicken embryo, suggesting that this compresses the surrounding tissue, causing the beak to develop (Fig. 13).

#### (b) A developmental hourglass

His compared embryos of different species stage‐by‐stage and showed that early embryos exhibit species differences in the size and distribution of the yolk and other factors:

… the most pressing question is how, from such different developmental starting points, do the similarities in subsequent [stages], and in the adult body, arise? (His, 1875, pp. 190–191)

His explained that, after these divergent early stages, the embryo undergoes folding into organ‐forming regions; the folding releases mechanical tensions in the tissues and so the embryo reaches an equilibrium state (His, 1875, p. 208). The embryos of different species become more similar in appearance, but nonetheless show the unique ‘physiognomy’ of their species. As development progresses further, the morphology of the embryos becomes increasingly divergent as they develop class characters (for example, feathers and beak in the chicken; His, 1875, p. 126). With this analysis, he described a pattern known today as the ‘developmental hourglass’ (Duboule, 1994), although His did not use that term.

### (7) Early brain development

**Fig. 14:**
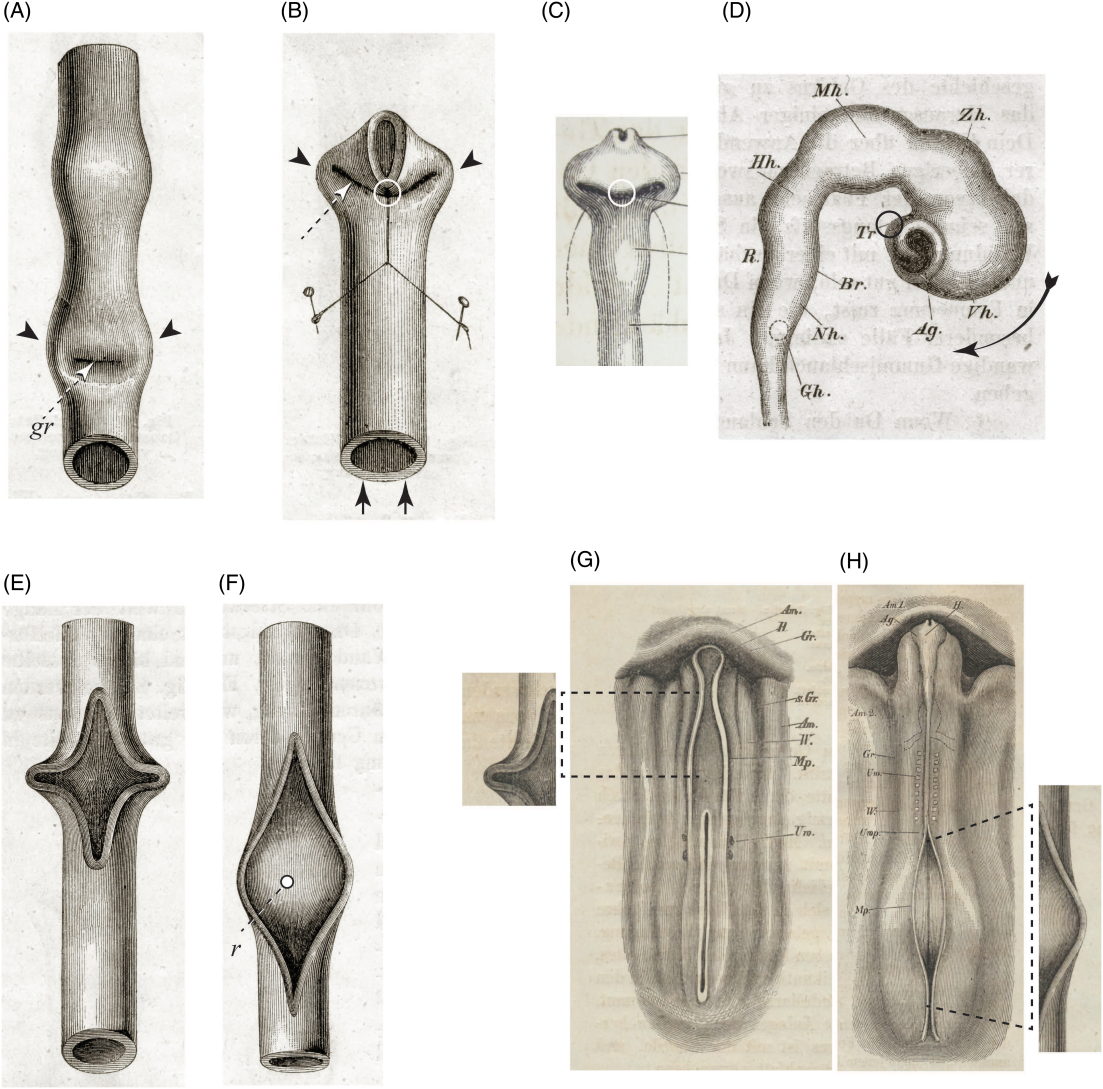
Rubber tubes used to model the morphogenesis of the cranial neural tube. Figures from His (1875). (A) Fig. 85. Flexion produces a transverse groove (*gr*) and two ‘ears’ (actually the optic Anlagen, arrowheads). (B) Fig. 86. When the tube is anchored at the white circle, then pushed in the direction of the black arrows, two ‘ears’, representing the optic Anlagen (arrowheads) and an oblique groove (white arrow) are formed. The anchorage point is the infundibulum attached to Rathke's pouch. (C) Fig. 82 (detail). Chicken embryo, 2 d incubation, ventral aspect of head end. The anchorage point in B is analogous to the infundibulum (white circle) in this embryo. (D) Fig. 84. Brain of chicken embryo, 3 d incubation, right lateral aspect. The infundibulum (black circle and *Tr*) represents the anchor point (white circle in B, C) which causes the bulging (curved arrow). (E) Fig. 87. A spindle‐shaped hole cut in the tube gapes to form a diamond‐shaped cavity when the tube undergoes convex flexure. (F) Fig. 88. With convex flexure of the slit tube, the floor of the aperture forms a transverse, saddle‐shaped ridge (*r*). (G) Fig. 14; chicken embryo, end of first day; dorsal aspect. The rubber model in E (shown in detail, left) resembles the shape of the neural Anlage where it passes from the head to neck (dashed lines). (H) Fig. 90. Chicken embryo, day 2, dorsal aspect. The open neural plate in the lumbar region resembles the slit tube in F (shown in detail, right).

**Fig. 15:**
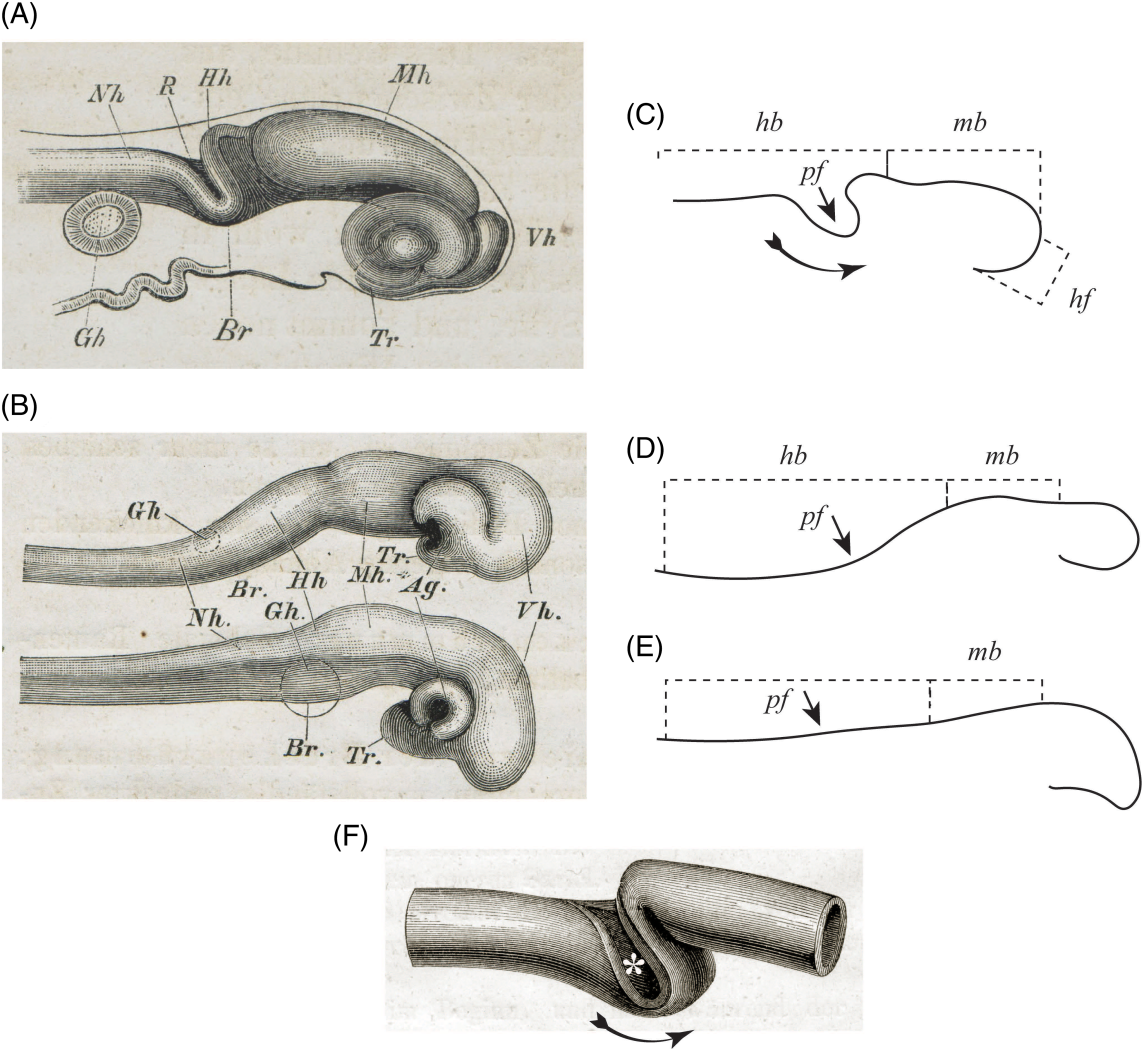
Comparison of brain development in three different vertebrates: (A) pike; (B) chicken (top) and frog (bottom). Figs 92, 101 and 102, respectively, in His (1875). (C, D, E) Our interpretation of His's text (His, 1875, pp. 107–108) for the pike, chicken and frog, respectively. The midbrain (*mb*) is relatively long in the pike. The pontine flexure (*pf*) is massively developed in the pike, weakly developed in the chicken and scarcely present in the frog. The massive development of the pike pontine flexure causes part of the hindbrain (*hb*) to appear subducted along the ventral aspect of the midbrain. *hf*, hook flexure or ‘Hakenkrümung’. (F) Model formed by pushing two ends of a slit rubber tube towards each other; subduction occurs (arrow) of part of the tube at the point of the slit (asterisk). This configuration resembles the pontine flexure in A, and the apparent subduction of part of the hindbrain on the ventral aspect of the midbrain (A, C; it is only ‘apparent’ because the midbrain is always rostral to the hindbrain).

Differential growth had been suggested as a causal factor in brain development by Reichert (1861) and later by His (1873a). In ‘*Our Bodily Form*’, His pursued this topic by analysing neural tube development in different vertebrates (His, 1875, pp. 93–118). His showed that rubber tubes, variously manipulated, can adopt shapes resembling the neural tube at different stages of development (Figs 14 and 15). The forms produced by the tubes bear a striking resemblance to the developing optic primordia, the rhomboid sinus, the infundibulum and the pontine flexure, implying that mechanical forces might play a part in neural tube morphogenesis.

#### (a) Flexures in the early neural tube influence the form of the adult brain

His noted that the midbrain in avian and osteichthyan embryos is relatively larger than in mammals (Fig. 15). He argued that these size differences influence the subsequent development of the brain and its adult form (he had made these arguments before: His, 1873a, pp. 331–332). He explained the relatively massive expanse of the human hemispheres in terms of the strong development of the pontine flexure, which in turn causes the hindbrain to be subducted under the ventral aspect of the midbrain. The brain is therefore shortened and more readily overgrown by the hemispheres (His, 1875, pp. 114–116).

#### (b) Each brain region ‘inherits’ a unique growth rate from its early primordium

According to His's earlier research (His, 1868b) the forebrain lies at the zenith of three growth gradients. Its relatively rapid growth continues in later stages, as though an autonomous growth rate is ‘inherited’ from its antecedent in the neurula. The forebrain grows so fast that it comes to bulge beyond the rostral tip of the notochord.

#### (c) Evolutionary differences in the relative size of the brain regions are established by heterochrony

His presented evidence that teleost embryos have a long midbrain compared to other vertebrates — and a correspondingly small forebrain. This size difference persists in the adult. One mechanism suggested by His to explain this phenomenon is an evolutionary change in developmental timing (His, 1873a, p. 331), which we would now call ‘heterochrony’ (Table S2). For example, the pontine flexure and ‘hook flexure’ (His's fourth primary flexure, see Fig. 15) are prominent in the developing salmon, and develop relatively early. By contrast, in the chicken at a similar stage, neither flexure is well developed.

### (8) After ‘*Our Bodily Form*’

In the years following the publication of ‘*Our Bodily Form’* , His's major publications included a landmark study of human developmental anatomy (His, 1880, 1882a, 1885). He also made important contributions to developmental neurobiology, proposing a scheme of neural tube organisation in which it is divided into ‘longitudinal zones’: the roof plate, floor plate, and side walls. The latter were subdivided into what he called alar and basal plates (His, 1888b, p. 350; Table S2). Orr (1887, p. 329) had also alluded to the longitudinal organisation of the neural tube (in *Anolis sagrei* and other lizards) but did not provide a zonation scheme.

## IV. THE INFLUENCE AND FATE OF HIS'S WORK

Prof. His has been led by his researches to adopt peculiar views concerning the causation of animal forms. These he has explained at some considerable length in his great work on the “Development of the Chick,” and elsewhere, but they have not met with very general acceptance (M.F., 1875)

### (1) An outstanding legacy in developmental neurobiology and descriptive embryology

‘In almost every branch of anatomy [His] has left his mark, and during the last thirty years no one has done so much to advance our knowledge of that subject. … there is no one who has exercised a more powerful influence in moulding anatomical thought in almost all branches of that subject.’ (Cunningham, 1906, pp. 1235–1236)

The contributions of Wilhelm His, Sr to neurobiology (Supplementary Note S3) and descriptive embryology were of great importance and he coined many anatomical terms still in current use (Table S2; Swanson, 2015, p. 17). His's contributions to neuron theory earned him two Nobel Prize nominations (Table S1). Minot (1890, p. 891) referred to His's ‘great’ monograph on chicken development (His, 1868b) and one reviewer described it as the finest work of its kind since von Baer (Anon., 1869). His's ‘*Anatomy of Human Embryos*’ (His, 1880, 1882a, 1885) is a foundational work in that field (O'Rahilly, 1979; Dupont, 2018, p. 2).

His's ideas influenced Weiss's ‘contact guidance’ theory of neuronal target‐finding (Weiss, 1955, p. 356), and his discovery of the neural crest is a landmark in the history of developmental neurobiology (Hall, 2008; Bronner & Simoes‐Costa, 2016; Dupont, 2018; Glover *et al*., 2018). On the other hand, his epi‐spinal spaces in the brain may be artefacts (Woollam & Millen, 1954), and his facial reconstruction of J. S. Bach is questionable (Supplementary Note S2).

… [His's] work on the development of the human hindbrain published in 1890 provided novel ideas that more than 100 years later form the basis for penetrating molecular investigations of the regionalization of the hindbrain neural tube … (Glover *et al*., 2018, p. S14)

**Fig. 16:**
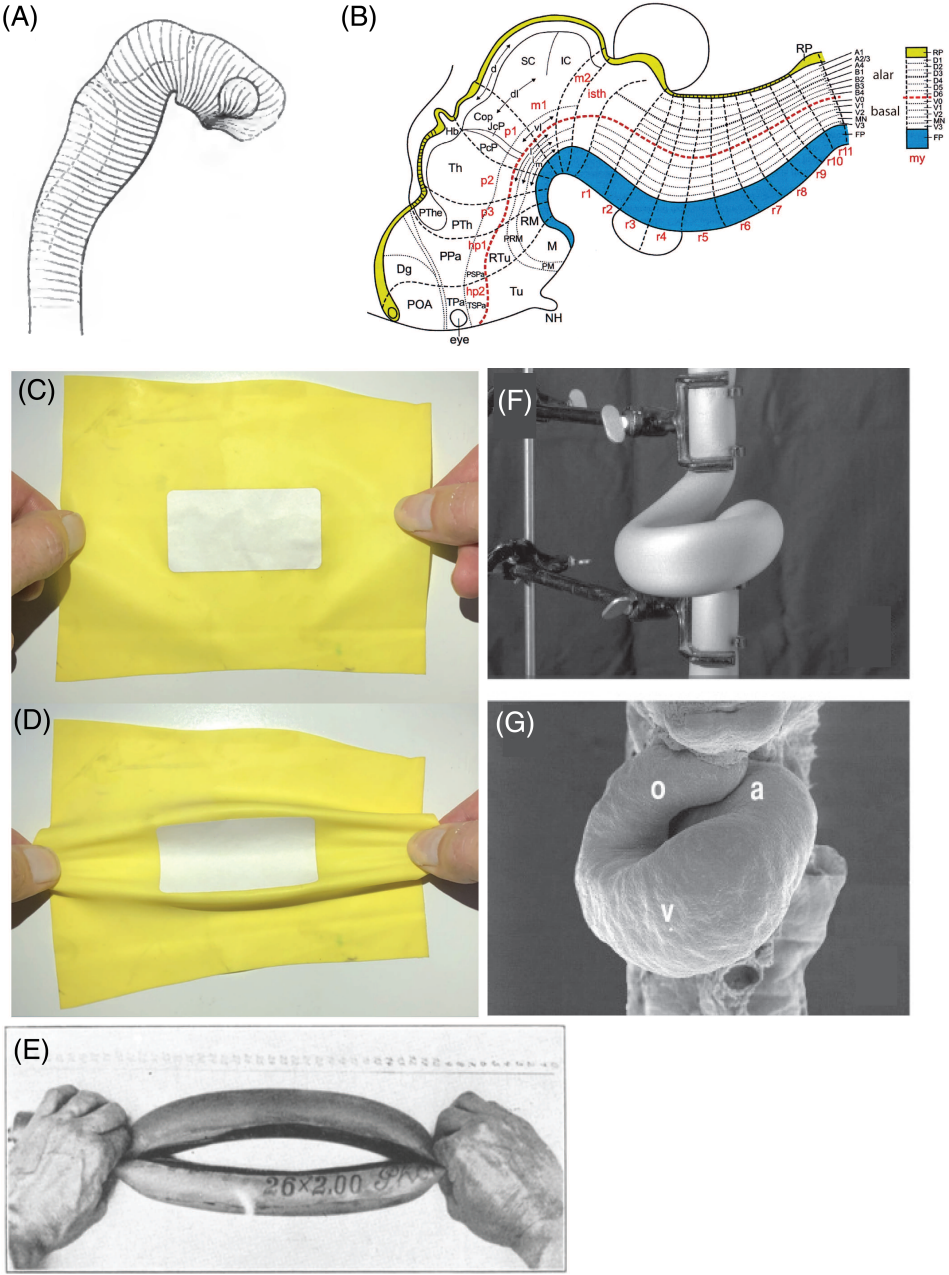
(A) His's model of longitudinal zones in the neural tube. Fig. 13 from His (1888b). (B) The ‘updated prosomeric’ model of brain organisation. Yellow, roof plate; blue, floor plate. Fig. 8 from Puelles (2018). (C, D) Folding along elasticity boundaries as a mechanism of early vertebrate morphogenesis (reconstructed by Dr. Fleury, based on fig. 16 in Fleury *et al*., 2015). A paper sticker is placed on a rubber sheet, thereby stiffening the central area. The stretched sheet buckles around the sticker, along boundaries between elastic and stiff regions. This demonstration is reminiscent of His' suggestion that the early embryo has variable elasticity and undergoes folding at unstable boundaries. (E) Vaihinger modelled the opening and closing of plant stomata by pushing and pulling a rubber tube (fig. 8 from Vaihinger, 1941). Courtesy of Universiteitsbibliotheek Utrecht. (F, G) Manipulation of a rubber tube (F) can create shapes resembling a scanning electron micrograph (G) of the developing cardiac tube of the chicken (fig. 5B, D from Männer, 2004).

His's longitudinal scheme of the nervous system was also highly influential, although Kingsbury (1920) argued that it may apply only to the truncal neural tube. Recent studies (reviewed by Puelles, Domenech‐Ratio & Martinez‐De‐La‐Torre, 1987; Glover *et al*., 2018; Puelles, 2018) suggest that His correctly identified the rostral extent of the alar‐basal boundary, but not that of the floor and roof plates (Luis Puelles, personal communication). The ‘updated prosomeric model’ is a synthesis of His's concept with current data; according to that model, the brain of all vertebrates can be divided into His's longitudinal zones, and several transverse segments or neuromeres (Puelles, 2018) (Fig. 16A, B).

In contrast to these impressive discoveries, His's theories of development and evolution did not have an enduring influence. This is partly because, as Mall explains, His ‘was never inclined to develop a school nor was he anxious to have pupils.’ (Mall, 1905, p. 150).

### (2) Failure of the parablast and concrescence theories

… after the fall of the parablast theory, the concrescence theory became [His's] problem child … he demonstrated concrescence using rubber and wax models very beautifully and clearly; however, rubber and wax are not the materials from which embryos are composed. (Rabl, 1897, pp. xiii–xiv)

The parablast and concrescence theories were, in the early days, highly influential. Van Beneden (1878, p. 51) referred to the ‘famous’ parablast theory as did Fick (1904, p. 187). Edwin Klebs developed a ‘parablastome’ theory to classify cancers according to their supposed parablastic origin (Klebs, 1889, pp. 573–574). As for concrescence, Rabl was sceptical but nonetheless described it as ‘ … one of the most important developmental theories about the structure of the vertebrate body…’ (Rabl, 1897, p. xii). Kopsch, also a sceptic, argued that: ‘the epoch‐making significance of His's concept consists in the extraordinarily simple way in which the embryonic development of vertebrates and invertebrates can now be explained by the same processes’ (Kopsch, 1904, p. 4). Concrescence was still cited in textbooks well into the 20th century.

Both theories, however, were ultimately shown to be false (Brauckmann, 2006). Cell‐labelling experiments were particularly important in ruling out concrescence (Keller *et al*., 1991). For details of the scientific evidence against these theories, see Supplementary Note S6. Picken (1956) argued that the failure of the parablast and concrescence theories may have tarnished His's scientific reputation.

### (3) His's mechanical developmental biology

Wilhelm His's approach to development is often called ‘mechanical’, a word that describes: (*i*) causal mechanisms; and (*ii*) the role of physical forces in development (Supplementary Note S7).

My attempts to introduce some elementary mechanical or physiological conceptions into embryology have not generally been agreed to by morphologists. (His, 1888a, p. 294)

His's mechanical developmental biology was important because it stimulated the study of developmental mechanisms, and because it provided a challenge to Haeckel's phylogenetic embryology (Supplementary Note S7). However, many scientists were critical because His inferred developmental mechanisms on the basis of histological studies and modelling, and not from *in vivo* experiments. Oscar Hertwig wrote: ‘ … I think more physiologically than His, who just wants to be the representative of a physiologically‐minded histology …’ (Hertwig, 1883, p. 124).

His's suggestion that differential growth is a major driver of gastrulation movements was contradicted by Rhumbler's studies suggesting that changes in cell shape and position, rather than growth, were important (see Table S4; Rhumbler, 1902, pp. 422–423). Subsequent studies have confirmed Rhumbler's view (Keller, 2002; Heisenberg & Bellaiche, 2013).

His suggested that neurulation was driven by pressure from tissues surrounding the neural primordium (Alexander Goette made similar suggestions; Goette, 1875, p. 186). However, Wilhelm Roux argued that the slightest variations in the parameters of the system would then cause the embryo to develop abnormally (Roux, 1895a, p. 242). Roux seems to be arguing that His's mechanism lacks what we now call developmental precision and robustness (see Kerszberg & Wolpert, 2007, pp. 205–206). Roux reported that surgically‐isolated neural primordia of chicken embryos in culture could still undergo normal neurulation movements, despite having been freed from any surrounding tissues (Roux, 1895a, pp. 247–248).

#### (a) *His and developmental mechanics (* Entwicklungsmechanik*)*

… [to His] we owe a whole series of causal deductions. However, we cannot deny that the applicability of this method to making causal deductions is very limited, and in many cases the conclusions thus obtained do not offer the certainty which is so desirable in the case of such fundamental questions. (Roux, 1895a, p. 30)

The developmental mechanics or *Entwicklungsmechanik* of Wilhelm Roux (Maienschein, 1991) was ‘mechanics’ not in the sense of physical forces, but in the sense of causal mechanisms (Roux, 1895a, p. 11; Rádl, 1909, pp. 517–522; Sander, 1991; Counce, 1994). Roux praised His for establishing a causal approach to development (Roux, 1895a, p. 244), but complained that His did not test his hypotheses experimentally (Roux, 1895b, p. 174). So, while His and Roux shared a common interest in mechanisms, Roux was an experimentalist, His was not. Because of this, Roux felt that His's explanations were insufficient. For example, he argued that His's model of differential growth in the embryo does not explain what causes the differential growth in the first place (Roux, 1895a, p. 147); similar criticisms were voiced by Spitzer (1886, p. 211).

#### (b) His and the modern discipline of mechanobiology

In ‘Membranes and cavities’, His speculated about the effects of physical forces and cell–matrix interactions in determining cell behaviour. The modern discipline of mechanobiology addresses such phenomena (Carter *et al*., 1998; Moore, Roca‐Cusachs & Sheetz, 2010; Iskratsch, Wolfenson & Sheetz, 2014; De, Hwang & Kuhl, 2015, p. v.; Jansen *et al*., 2015; Paluch *et al*., 2015). Mechanobiology is a recent discipline, one of the earliest mentions of which is in van der Meulen, Beaupre & Carter (1993).

Cells can sense forces applied to the extracellular matrix, and the transduction of these forces can influence stem cell behaviour (Engler *et al*., 2006; Discher, Mooney & Zandstra, 2009). There is increasing evidence that these phenomena are important in development — as His had suggested. The application of biomechanics to development was called ‘embryo mechanics’ by Davidson, who cites ‘*Our Bodily Form*’ as an early work in the field (Davidson, 2011, 2017).

Physical forces are important in modulating cell behaviour in the developing nervous system (Van Essen, 1997; Hilgetag & Barbas, 2005). Some recent authors have found merit in His's rubber tube models of neural tube development (Sheesley *et al*., 2014; Edelman *et al*., 2016). Others have presented evidence that mechanical forces play an important role in the formation of gyri and sulci on the surface of the cortex (Tallinen *et al*., 2016). Physical forces established by blood flow patterns (haemodynamics) are important in the development of the cardiovascular system (reviewed by Stekelenburg‐de Vos *et al*., 2003; Groenendijk *et al*., 2005; Andres‐Delgado & Mercader, 2016; Poelmann & Gittenberger‐de Groot, 2018). Fusion of the cardiac tubes at the midline may be influenced by tensile forces in the endoderm (Varner & Taber, 2012).

At least one recent paper on mechanobiology cites ‘*Our Bodily Form*’ as an early work relevant to the field (Keller, Shook & Skoglund, 2008). But other reviews of the history of mechanobiology make no mention of His (Iskratsch *et al*., 2014; Wall *et al*., 2018), suggesting that their authors do not regard him as a major influence on the field.

#### (c) The viscoelasticity of embryos

The dispute between His and Haeckel over the elasticity of the embryo appears to have been resolved in His's favour. Elasticity has been demonstrated in early chicken (Agero, Glazier & Hosek, 2010) and *Xenopus laevis* (Zhou, Kim & Davidson, 2009) embryos. One current model sees embryos as viscoelastic, i.e. having both fluid and elastic properties. Viscoelasticity varies at different locations in early chicken embryos (Marrese *et al*., 2019). Fleury *et al*. (2015) modelled the viscoelastic properties of early embryos, and how they are deformed by physical forces. In one experiment, the authors applied forces to a sheet of rubber with a paper sticker fastened to it (Fig. 16C, D) and showed that boundaries between regions differing in elasticity were crucial hinge‐points (Fleury *et al*., 2015).

### (4) His and evo‐devo

“… comparative developmental mechanics” is a science that lies in the future (Roux, 1895a, p. 441)

Evo‐devo (evolutionary developmental biology) is a discipline which came to prominence following the publication of *Ontogeny and Phylogeny* by Stephen Gould (Gould, 1977) and the cloning of homeobox genes in *Drosophila* and mammals in 1984 (see Richardson, 2009). One of the aims of evo devo is to explain phenotypic evolution in terms of evolutionary changes in developmental mechanisms (Raff, 2000; Swalla, 2002; Gilbert, 2003; Hall, 2003; Tickle & Urrutia, 2017). His's work has had little influence on the modern discipline of evo‐devo; Gould is the only reviewer among those cited above who mentions His's work. Nevertheless, we argue that ‘*Our Bodily Form*’ contains many ideas relevant to this field.

#### (a) Ontogenetic allometry

Mehnert, inspired by His's growth law and organ‐forming regions, suggested that organ primordia might function as independent growth centres (Mehnert, 1898; pp. 76–77). Today, such phenomena are considered under the rubric of ontogenetic allometry: changes in the relative dimensions of an individual or its parts during development (Gould, 1966; pp. 587, 601–602). Eduard Hagenbach's calculations, published in His (1868b), are an early example of the mathematical modelling of ontogenetic allometry; His's morphometric analysis of six amniote embryos is another (see Figs 10, 11, 12).

His's suggestion that the massive growth of the eye in the chick embryo causes the beak to develop, is an example of ontogenetic allometry (Fig. 13; His, 1875, pp. 204–206). Haeckel (1875, pp. 27–28) argued that it could just as easily be the case that the beak drives development of the eye. His's idea, however, is more plausible, given that the eye develops much earlier than the beak (a point suggested to us by Robert Poelmann). Discussions of beak morphogenesis are interesting in the light of a recent study of the variations in beak shape among Darwin's finches (*Geospiza* spp.; Abzhanov *et al*., 2004) which found that the timing of *Bmp4* expression in the upper beak primordium might explain the ontogenic allometry of the beak.

#### (b) The problem with phenetic comparisons

His's comparison of six amniote embryos (Figs 10, 11, 12) is problematic for many reasons. The stages are not equivalent (Fig. 10; Spitzer, 1886, p. 152), and only external characters are considered. Additionally, His's drawing (see Fig. 10) of the deer embryo is problematic because of its tendentious depiction of the acropodia (Richardson & Keuck, 2001). Another major objection to His's comparison is that it is a phenetic comparison — a pre‐cladistic approach to grouping organisms based on their overall similarity (Cain & Harrison, 1958, 1960; Sneath & Sokal, 1962; Vernon, 1988; Richardson *et al*., 2001; Rossello‐Mora & Amann, 2001). As Spitzer (1886, p. 155) put it, His's comparisons were based on the ‘impression of the aesthetic similarity’ of embryos.

#### (c) The ‘developmental hourglass’

The influential developmental ‘hourglass’ (egg‐timer) model in evo‐devo is based on phenetic comparisons and on transcriptome analyses. It is a statement of the gross similarity of embryos at different stages of development. It envisages convergence of the external morphology of young stages towards a mid‐embryonic, conserved stage; and then divergence in morphology as embryos progress to postembryonic stages (reviewed by Duboule, 1994; Richardson & Keuck, 2002). The relatively high similarity of these mid‐embryonic stages among different species is suggested to reflect the conservation of gene regulatory networks active at those stages (Duboule, 1994; Prud'homme, Gompel & Carroll, 2007).

Von Baer had noted the similarity of early vertebrate embryos, and their subsequent divergence as they develop (von Baer, 1828, p. 221). He argued that all animals develop from a phenotypically similar early stage (von Baer, 1828, pp. 223–224): a vesicle, which Haeckel later equated with his ‘blastula’ (Haeckel, 1877, p. 153). Because von Baer's pattern of divergence represents only the upper part of the developmental hourglass, it has been called the developmental ‘funnel’ (Abzhanov, 2013).

It was His who first identified the hourglass pattern and Keibel (1906, p. 172) confirmed this, although neither used the term ‘developmental hourglass’. Recent transcriptomic studies have largely confirmed the existence of a middle stage in development where transcriptomes are relatively enriched with conserved developmental genes (Quint *et al*., 2012; Tena *et al*., 2014; Drost *et al*., 2015, 2016; Cridge, Dearden & Brownfield, 2016; Marlétaz *et al*., 2018; Yanai, 2018; discussed by Richardson, 2012).

#### (d) Embryonic characters in phylogenetic studies

His believed that ‘younger’ embryos show class, genus, species and individual traits; and he tried to group the species based on measurements of their bodily proportions (see Fig. 12; His, 1875, p. 201). Spitzer argued that this could potentially be used in a new classification of animals based on embryonic characters (Spitzer, 1886, pp. 154–156) but was sceptical about whether embryos could provide as much information as adult stages (Spitzer, 1886, p. 155). He also argued that using His's approach would mean that human and chick embryos, on the basis of their large heads, would be more closely related to one another than to the other species considered; a conclusion that would be inconsistent with known phylogenies.

#### (e) Quantitative methods in comparative embryology

It was, perhaps, in his views on the importance of measurement for the understanding of morphogenetic processes that His displayed the greatest originality. (Picken, 1956, p. 1163)

His's comparison of six amniote embryos, in which he applied morphometrics to comparative embryology (see Fig. 12) is a landmark in the history of evolution and development. It attempts to supplement phenetic statements about embryonic similarity with quantitative data on the relative dimensions of embryonic regions. However, His's data were limited to three regions in six embryos at a single stage with no replicates. Modern quantitative approaches to comparative embryology are found in the study of heterochrony (e.g. Jeffery *et al*., 2005; de Jong *et al*., 2009; reviewed by Klingenberg, 1998; Keyte & Smith, 2014), allometry (Klingenberg, 2016), and developmental transcriptomics.

#### (f) Coordinate systems, positional information and gradients

Some of the concepts that His explored have more recently become part of our understanding of developmental mechanisms. These include such concepts as coordinate systems, positional information gradients and cell–cell signalling. However, His's work on these concepts did not lead to lasting insights, and later interest in those same subjects appears to be independent of any influence or knowledge of His's work.

His used a coordinate system to map loci of differential growth, and growth gradients, in the embryo (His, 1875, pp. 121–122; His, 1867a, p. 619; His, 1868b, p. 185). Later, D'Arcy Thompson used *x–y* coordinates to map body plans onto rubber sheets; he then stretched the sheets to model the effects of differential growth (Thompson, 1945, p. 1087). Driesch also considered development in terms of coordinates: ‘what actually will happen in each of the blastula cells in any special case of development experimentally determined depends on the position of that cell in the whole, if the ‘the whole’ is put into relation with any fixed system of co‐ordinates … ’ (Driesch, 1908, p. 80; see also Driesch, 1891, pp. 28–29). Another example of the use of coordinate systems in anatomy is in its application to brain structure (Nieuwenhuys & Puelles, 2016).

His postulated that each organ primordium adopts its own growth law depending on its spatial relationships with its neighbours (His, 1875, p. 83). This is a statement of how a rapidly growing tissue might apply mechanical force to its neighbours. An analogous suggestion was made by Schwendener in his positional theory of phylotaxis based on mechanical influences (Schwendener, 1878).

His proposed that cells and tissues might influence each other *via* the secretion of chemicals (His, 1865b, p. 33). However, he considered gradients not in terms of concentration gradients in chemicals, but as growth gradients. The concept of growth gradients that His proposed does not appear to have influenced the development of gradient theory by Child (1928), Runnström (1914) and others (reviewed by Spemann, 1938, pp. 318–345). Spemann stated that the concept of developmental gradients ‘as far as I know, was first proposed and confirmed by Th. Boveri’ (Spemann, 1938, p. 318). Gradients of morphogens became a key concept in Wolpert's positional information model (Wolpert, 1969), although he later modified this view (Kerszberg & Wolpert, 2007; Wolpert, 2009).

### (5) Modelling

His used a variety of physical materials in order to model developmental processes (Table S4), and to make 3‐D models for teaching (Hopwood, 1999, 2002, 2012). Haeckel poured scorn on His's ‘comical’ models that used rubber tubes and postal envelopes (Haeckel, 1875, pp. 26–27). However, many developmental biologists have used models of various kinds (e.g. Fig. 16; Table S4): Rhumbler (1902) made a model of the blastula out of corset stays, Vaihinger (1941) used rubber tubes to model the biomechanics of stomatal opening, and Männer (2004) applied forces to a rubber tube to model cardiac looping (Fig. 16C–G; see also Taber, Lin & Clark, 1995).

### (6) The fate map and organ‐forming regions

This view was formerly introduced into embryology by His as the principle of organ‐forming germ regions; it shows affinity to the views of Weismann and Roux (Maas, 1903, p. 65). (The 'view' referred to was that different regions of the early embryo develop into distinct parts of the adult)

#### (a) The fate map

One of His's research goals was to establish an exact topography of the chicken embryonic disc (His, 1877a, p. 112). His's map of organ‐forming regions (Fig. 8) is the earliest fate map known to us. However, it was entirely speculative; true fate maps would only emerge from cell‐labelling experiments (Goodale, 1911; Smith, 1914; Vogt, 1929; Psychoyos & Stern, 1996). When Rawles compared His's fate map to her own experimental results, she concluded: ‘while a certain striking similarity exists between the theoretical concepts of His and the present experimental analysis with regard to some points, His's scheme is based on too simple a ground to justify detailed comparison.’ (Rawles, 1936, p. 312).

#### (b) Organ‐forming regions and mosaicism

His's theory of organ‐forming regions was influential in discussions of mosaic development (Supplementary Note S8). Roux wrote: ‘If a whole arises from several or many independently differentiating parts, it is assembled, like a mosaic, from individual parts that have been formed; I have called this kind of development a “mosaic work”’ (Roux, 1895a, p. 821). Roux noted that ‘His’ principle of organ‐forming germ‐regions … only has a causal meaning insofar as it designates the locations of the resultant formation of manifold interactions' (Roux, 1895a, p. 825).

Weismann Parker & Rönnfeldt (1893) state that His's theory of organ‐forming regions was the first, albeit imperfect, expression of the predestination of cells. They argue that His's theory correctly suggests that the ‘differentiating principle’ of development lies within the cell itself and not in external influences (Weismann *et al*., 1893, pp. 134–135). His's concept also influenced Wilson's ‘germinal localisation’ theory (reviewed by Kollmann, 1904; Wilson, 1904). For more recent opinions on regulation and mosaicism, see Boiani *et al*. (2019) and Stern (2006).

#### (c) Organ‐forming regions as fields

The organ‐forming regions of His are growth centres. But he did also talk in terms of ‘fields’ demarcated by ‘boundary markers’ in the early chick embryo (His, 1875; pp. 45–46) as, later, did Wolpert (1969; see also Jacobson, 1983, p. 1036). The concept of morphogenetic fields was elaborated first by Alexander Gurwitsch and then by Paul Weiss (Beloussov, Opitz & Gilbert, 1997). Today, developmental fields are considered to be regions undergoing morphogenesis or pattern formation (Wolpert, 1969; De Robertis, 2009).

### (7) Technical innovations

The technic of investigation made little progress, and it is in this particular that His made a change. He had prepared himself by a study of the embryology of the chick and in connection with this study had worked out a successful technic; he fixed his embryos, he had constructed an apparatus for section cutting which, primitive as it now seems, led the way to our modem microtomes, and he had thought out a method of reconstruction which has been gradually improved, especially by Born, into a method that is now absolutely indispensable in embryological investigation. (Keibel & Mall, 1910, p. xv)

His's microtome was certainly not the first section cutter. In 1770, Hill described a ‘cutting engine’ designed by Cummings for the sectioning of woody plant tissue (Hill, 1770). Adams' microtome had a micrometer‐screw advance (Adams & Kanmacher, 1798, p. 128; Chvátal, 2017). Chevalier used the name ‘microtome’ for such devices (Chevalier, 1839, p. 192). A microtome for the sectioning of animal tissue was developed by Oschatz in Purkyně's lab, long before the invention of paraffin embedding (Oschatz, 1844; Chvátal, 2017).

The sections that His cut routinely were 50 μm in thickness — far thicker than the 5–7 μm sections common today. His was still using his microtome in his studies of human embryos in the 1880s. But it is likely, from reading his text and tables (His, 1880, pp. 118, 137; His, 1885, pp. 7–9) that he used even thicker sections (up to 200 μm) for particuarly large embryos and fetuses. Hermann Fol suggested that this was too thick, and that it may have led to errors in interpretation (Fol, 1884, p. 381; His, 1885, p. 11 n.). Hochstetter also questioned the reliability of His's interpretations and criticised the poor quality of his human embryos (Hochstetter, 1919; pp. 1, 9). On these grounds, Hochstetter argued that the fame and influence of His's publications on human embryology may not be entirely deserved. Eventually, His abandoned his own microtome in favour of the Altmann‐Schanze microtome which yielded sections of 20–25 μm (Anon., 1885, pp. 547–548; His, 1885, p. 7).

## V. CONCLUSIONS

1. His was a developmental anatomist and technical innovator. Much of his descriptive work is now part of the fabric of embryology, anatomy and neurobiology.
2. His's publications were full of new ideas about evolution, development and mechanical developmental biology. Few of these ideas had an enduring influence, perhaps because His never cultivated a school of followers. Another reason is that these theories were speculations based solely on histological data and modelling. Experimental embryology later would provide more persuasive data and more robust theories.
3. Despite the criticism that they received, many of His's ideas have analogues in modern biology. They are only analogues – independent occurrences of the same idea. But the study of their origin and fate provides a fascinating case study in the history of scientific ideas.

## Supporting information


**Fig. S1.** Keys to sitters in the group photographs shown in Fig. 2 of the main text.
**Fig. S2.** Bibliographic analysis of the research themes of Wilhelm His.
**Table S1.** Wilhelm His, Sr. – selected biographical landmarks.
**Table S2.** Glossary of terms and concepts relevant to this review.
**Table S3.** Opinions of earlier researchers on the origins of the peripheral ganglia and the nephric duct.
**Table S4.** Models used by His and others to explain and understand developmental processes.
**Table S5.** Table of Contents of ‘*Our Bodily Form*’ (His, 1875) with chapter summaries.
**Supplementary Note S1.** Additional information on the oil painting of Wilhelm His shown in Fig. 1D.
**Supplementary Note S2.** Contributions of Wilhelm His Sr. to anthropology and forensic craniofacial reconstruction.
**Supplementary Note S3.** Contributions of Wilhelm His Sr. to developmental neurobiology.
**Supplementary Note S4.** The conflict between Wilhelm His and Ernst Haeckel.
**Supplementary Note S5.** Other polemics and disputes.
**Supplementary Note S6.** Scientific opinion on the parablast and concrescence theories.
**Supplementary Note S7.** His's ‘mechanical’ developmental biology: its meaning and its reception.
**Supplementary Note S8.** Mosaic *versus* regulatory development.
