# Supplementary material for: The revolutionary developmental biology of Wilhelm His, Sr
Source: Biol Rev Camb Philos Soc. 2022 Feb 1;97(3):1131–60. doi: 10.1111/brv.12834 (PMC9304566; doi:10.1111/brv.12834)
Supplement: Supplementary file 1 — Fig. S1. Keys to sitters in the group photographs shown in Fig. 2 of the main text. Fig. S2. Bibliographic analysis of the research themes of Wilhelm His. Table S1. Wilhelm His, Sr. – selected biographical landmarks. Table S2. Glossary of terms and concepts relevant to this review. Table S3. Opinions of earlier researchers on the origins of the peripheral ganglia and the nephric duct. Table S4. Models used by His and others to explain and understand developmental processes. Table S5. Table of Contents of ‘Our Bodily Form’ (His, 1875) with chapter summaries. Supplementary Note S1. Additional information on the oil painting of Wilhelm His shown in Fig. 1D. Supplementary Note S2. Contributions of Wilhelm His Sr. to anthropology and forensic craniofacial reconstruction. Supplementary Note S3. Contributions of Wilhelm His Sr. to developmental neurobiology. Supplementary Note S4. The conflict between Wilhelm His and Ernst Haeckel. Supplementary Note S5. Other polemics and disputes. Supplementary Note S6. Scientific opinion on the parablast and concrescence theories. Supplementary Note S7. His's ‘mechanical’ developmental biology: its meaning and its reception. Supplementary Note S8. Mosaic versus regulatory development. [file BRV-97-1131-s001.docx]

**Supporting Information**

| **CONTENTS** |  |
| --- | --- |
| **Fig. S1.** Keys to sitters in the group photographs shown in Fig. 2 (main text). | 2 |
| **Fig. S2**. Bibliographic analysis of the research themes of Wilhelm His. | 4 |
| **Table S1**. Wilhelm His, Sr. – selected biographical landmarks. | 5 |
| **Table S2**. Glossary of terms and concepts relevant to this review. | 8 |
| **Table S3**. Opinions of earlier researchers on the origins of the peripheral ganglia and the nephric duct. | 15 |
| **Table S4**. Models used by His and others to explain and understand developmental processes. | 17 |
| **Table S5**. Table of contents of ‘*Our Bodily Form*’ (His, 1875) with chapter summaries. | 20 |
| **Supplementary Note S1**. Additional information on the oil painting of Wilhelm His shown in Fig. 1D of the main text. | 22 |
| **Supplementary Note S2**. Contributions of Wilhelm His Sr. to anthropology and forensic craniofacial reconstruction. | 23 |
| **Supplementary Note S3**. Contributions of Wilhelm His Sr. to developmental neurobiology. | 24 |
| **Supplementary Note S4**. The conflict between Wilhelm His and Ernst Haeckel. | 25 |
| **Supplementary Note S5**. Other polemics and disputes. | 26 |
| **Supplementary Note S6**. Scientific opinion on the parablast and concrescence theories. | 28 |
| **Supplementary Note S7**. His’s ‘mechanical’ developmental biology: its meaning and its reception. | 29 |
| **Supplementary Note S8**. Mosaic versus regulatory development. | 31 |

# Fig. S1. Keys to sitters in the group photographs shown in Fig. 2 (main text).

Based on information printed at the foot of the original photographs. Our assignment of names should be considered provisional, especially for the Tübingen photograph (B) where the participants were not standing in an orderly formation.

**(A)** Anatomische Gesellschaft, Meeting, Basel, 1895.


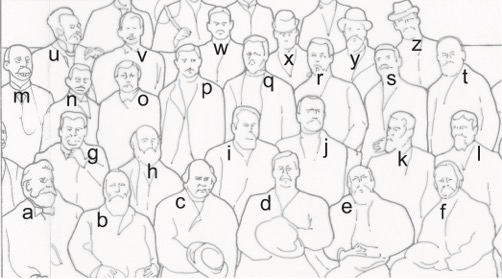


| **Row** (from front to back) | **4** | u  Paul Eisler | v  Heinrich von Eggeling | w  Ernst Göppert | x  Henry Bargman Pollard? | y  Friedrich Maurer | z  Hanson Kelly Corning |  |  |
| --- | --- | --- | --- | --- | --- | --- | --- | --- | --- |
|  | **3** | m  Franz Keibel | n  Ernst Gaupp | o  Hermann Klaatsch | p  Erich Kallius | q  Georg Thilenius | r  Ernst Mehnert | s  Dimitrie Gerota | t  August Froriep |
|  | **2** | g  Walther Felix | h  Moritz Nussbaum | i  Hector Leboucq | j  Adolphe Nicolas | k  Auguste Eternod | l  Friedrich Kopsch |  |  |
|  | **1** | a  Gustav Schwalbe | b  Wilhelm Waldeyer | c  Albert Kölliker | d  Friedrich Merkel | e  Stieda | f  Wilhelm His, Sr. |  |  |

**(B**) Anatomische Gesellschaft, Meeting, Tübingen, 1899.


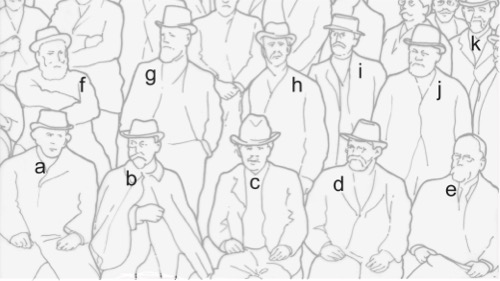


| **Row** (from front to back) | **3** |  |  |  |  | k  Johan Hultkrantz |
| --- | --- | --- | --- | --- | --- | --- |
|  | **2** | f  Guglielmo Romiti | g  Édouard Van Beneden | h  Wilhelm His, Sr. | i  Paul von Baumgarten | j  Gustav Schwalbe |
|  | **1** | a  Albert von Kölliker | b  Walther Flemming | c  Gustaf Retzius | d  Friedrich Merkel | e  August Froriep |

# Fig. S2. Bibliographic analysis of the research themes of Wilhelm His.

Based on the bibliography in Fick (1904), but with non-scientific works excluded, redundant citations resolved, and use of our own categories. His’s diverse range of research interests was not unusual for a biologist at that time. See, for example, the very wide-ranging publications of von Baer (von Baer & Oppenheimer, 1986), Darwin (in Freeman, 1977) and Kölliker (1899).





# Table S1. Wilhelm His, Sr. – selected biographical landmarks.

For major biographies of of Wilhelm His, see Fick (1904) and Waldeyer (1904); and for His’s memoirs see His (1903). Wilhelm His Jr. looks back on his father’s life in His (1931). For other works with biographical information, see Bertolini (1965), Hildebrand (2005), Kästner (2005), Kollmann (1904), Mall (1905), Müller & O'Rahilly (1986), O'Rahilly & Müller (1988), Picken (1956), Richards (2008), Spalteholz (1906), Tubbs *et al*. (2009), Waldeyer-Hartz (1921) and Wendler & Rother (1982) and the many obituaries (Anon., 1904*a*; Anon., 1904*b*; Cunningham, 1906; Dixon, 1904; Marchand, 1905; Rawitz, 1904; Voit, 1905). Waldeyer (1921) shares reminiscences about his close friendship with His.

The study of Wilhelm His and his work is hindered by the fact that his Anatomical Institute, and many other University buildings, were destroyed in the Allied bombing of Leipzig in the Second World War (Lehmstedt, 2003). For the history of the Anatomical Institute see Rabl (1909*a*,*b*).

Perhaps His’s most notable student was Franklin Mall (Sabin, 1934). Mall started in His’s lab in 1884 to work on the thymus. Mall thought that the thymus originated from the endoderm but His thought it came from the ectoderm (of the third gill cleft; Sabin, 1934; Fig. 3C in our main text). Mall then went to study in Ludwig’s lab (1856–1885) on His’s advice. Charles Minot was not a student of His but was in Leipzig and studied under Carl Ludwig and Rudolph Leuckart (Minot, 1897, p. viii). Minot was in Ludwig’s laboratory from 1873 to 1875 (Lewis, 1916, pp. 141–142).

| **Year** | **Key events** | **Source** |
| --- | --- | --- |
| 1831 | Born July 9th, Basel, Switzerland | His (1903) |
| 1849–1854 | Medical student, University of Basel | His (1903) |
| 1849–1850 | To His’s dismay, he missed attending lectures by Alexander Ecker in Basel; Ecker had just moved to Freiburg. Semesters 2 and 3, attends lectures in Berne from Gustav Valentin. | His (1903) |
| 1850–1852 | Semesters 4–6, Berlin. Teachers include Johannes Müller and Robert Remak. His found Müller to be an inspirational teacher. The lectures from Remak on embryology, and the visits afterwards to the embryology laboratory in Remak’s home, had a significant influence on His’s later research.  His reads a paper by Rudolph Virchow that opened extensive discussion of the ‘connective tissue question’. His was so excited by that paper that he went to Würzburg, where Virchow lectured. For more on the connective tissue question, see His (1903, pp. 37–39). | His (1903); Rabl, (1909*b*); Virchow (1852) |
| 1852–1861 | [Carl Ludwig’s ‘*Textbook of Human Physiology*’ published] | Ludwig (1852, 1856) |
| 1852–1853 | His goes to Würzburg at the beginning of semester 7. Virchow encouraged His to undertake a research project on the structure of the cornea. His has some interaction with Kölliker, but the timing of his semester means that he misses his lectures and famous histology course. He reads many of Hermann Lotze’s works with great interest (His, 1903, p. 36) including his ‘*General Physiology*’ which includes a chapter on the development of form (Lotze, 1851). | His (1903) |
| 1854 | First scientific publication: ‘The Structure of the Cornea’. Presented 1853, published 1854. Followed in 1856 by a second on the same topic. | His (1854, 1856) |
| 1854 | After excursions to Prague (1853) and Vienna (1854) His returns to Basel and passes the *Doktorexamen* summa cum laude. | His (1903, 1931); Rabl (1909*b*) |
| 1855–56 | To Paris with the hope of interacting with Claud Bernard and attending his lectures. Unfortunately, Bernard wasn’t often in the laboratory during the winter, and his lectures only started when His was leaving. | His (1903) |
| 1856 | Habilitation (post-doctoral dissertation): an oration on cells and a lecture each on histology and histopathology | Rabl (1909*b*) |
| 1857–1872 | Professor of Anatomy and Physiology at the University of Basel | His (1903, 1931) |
| 1859 | [Darwin: *On the Origin of Species*…, first English edition published] | Darwin (1859) |
| 1860 | [Bronn: First German edition of Darwin, *On the Origin of species*…] | Bronn (1860) |
| 1865 | First developmental study: ‘On the Structure of the Mammalian Ovary’ | His (1865*a*) |
| 1865 | ‘Membranes and Cavities’ | His (1865*b*) |
| 1866 | [*In ‘General Morphologie*’ Haeckel states his version of ‘recapitulation’ theory and presents a mechanism of evolution and development, based on heredity and adaptation]. | Haeckel (1866*a*) |
| 1865 | [Carl Ludwig appointed Professor of Physiology at Leipzig] | Fick (1895) |
| 1868 | Monograph on early chicken development | His (1868*b*) |
| 1868 | [Haeckel publishes first edition of ‘*Natural History of Creation*’] | Haeckel (1868) |
| 1869 | Rektor, University of Basel | His (1931); https://unigeschichte.unibas.ch/materialien/rektoren/ |
| 1872–1904 | Professor of Anatomy at the University of Leipzig | His (1903, 1931) |
| 1872 | [Haeckel publishes ‘*Calcareous Sponges*’] | Haeckel (1872) |
| 1874 | [Haeckel publishes first Edition of ‘*Anthropogenie: The History of Man*’ which includes criticism of His’s (1868*b*) monograph on chicken embryology] | Haeckel (1874) |
| 1875 | [Alexander Goette publishes ‘*Development of the Fire-bellied Toad*’] | Goette (1875) |
| 1875–1904 | Editor (with Wilhelm Braune) of *Archiv für Anatomie und Entwickelungsgeschichte*. Volumes 1 (1876) and 2 (1877) were published under the title *Zeitschrift für Anatomie und Entwicklungsgeschichte*. | From the front matter of the relevant journals; see also the Editors’ announcement of the name change, end of *Zietschrift* Vol 2, 1877. |
| 1875 | Unsere Körperform (‘*Our Bodily Form*’) | His (1875), frequently mis-cited as 1874. |
| 1875 | [Haeckel publishes ‘*Aims and Methods*’ a response, in part, to ‘*Our Bodily Form*’ of His] | Haeckel (1875) |
| 1878, 1888, 1889 | Dean of Medical Faculty, University of Leipzig | Fick (1904) |
| 1882 | Rektor, University of Leipzig | https://www.archiv.uni–leipzig.de/geschichte/universitatsgeschichte/personen/rektoren-der-universitat/ (accessed 23-11-2017) |
| 1895 | Anatomy Congress at Basel, 17–20 April, with meetings to discuss the ‘*Basel Nomina Anatomica*’ | His (1895*b*) |
| 1900–1901? | Nomination by Gustaf Retzius of Wilhelm His (along with Ramon y Cajàl and Albert von Kölliker) for the 1901 Nobel Prize in Medicine or Physiology. Motivation: ‘Work in embryology, especially on the neuroblast, and the structure of the protoplasm.’ | https://www.nobelprize.org/nomination/redirector/?redir=archive/ |
| 1903–1904? | Nomination by August Rauber of Wilhelm His (with Gustaf Retzius and Ramon y Cajàl) for the 1904 Nobel Prize in Medicine or Physiology. Motivation: ‘Important publications in embryology and organogenetics.’ | ibid. |
| 1904 | Died May 1st from cancer of the stomach and liver | Fick (1904); His, (1931); Spalteholz, (1906); Waldeyer-Hartz (1921); His, (1931, p. 55). |
| 1943 | December 4th, Anatomical Institute of the University of Leipzig destroyed by fire started by Allied bombing. Some materials were rescued but most of His’s collections were lost. Other materials from His’s laboratory are currently preserved in the Anatomisches Museum, Basel. The Human Developmental Anatomy Center, National Museum of Health and Medicine, Maryland, USA, currently has one human embryo formerly in the collection of Wilhelm His (Elizabeth Lockett, pers. comm.). | Hehl *et al*. (2010, pp. 317–318); Lehmstedt (2003, pp. 146–149); Bertolini (1965); Kurz (1992) |

# Table S2. Glossary of terms and concepts relevant to this review.

In addition to serving as a glossary for the main text, this table provides examples that illustrate His’s contributions to biology. We have set these contributions in context by listing some of the terms and concepts of other scientists. Some terms in this table are in such common usage that we do not need to provide a definition; we have included these terms only to show their origins in the literature. For additional authorship information see Assmann (1847) for comparative anatomy, and Dejerine (1895) and Swanson (2015) for neuroanatomy. For Wolff, see also the German translation (Wolff, 1812). *We list the earliest author we know for these terms. If any reader discovers a prior usage, we would be pleased to be informed.

| Term or concept | Meaning | Earliest author* | Reference | Notes |
| --- | --- | --- | --- | --- |
| adenoid tissue | The follicular (reticular) tissue of the lymph glands, thymus and intestinal wall | His | His (1862, p. 423 n.) |  |
| alar lamina, basal lamina | Dorsal and ventral part, respectively, of the lateral wall of the truncal neural tube | His | His (1888*b*, p. 350) | Flügelplatte and Grundplatte, respectively |
| alecithal, telolecithal, centrolecithal | Statements of yolk quantity and distribution in eggs | Balfour | Balfour (1880, p.90) | Balfour credits Lankester for suggesting these names (Balfour, 1880, p. 90, n. 1) |
| allantois |  | Aristotle? | Thompson (1910) | Described in the chicken egg (‘a membrane’) but not named (Thompson, 1910, p. 561b n. 4); Thompson doubts the authenticity of this part of the text |
|  |  | Malpighi | Malpighi (1673) | Not named as such, but illustrated (Malpighi, 1673, Fig. 19G) |
| amnion |  | Empedocles |  | According to Rufus of Ephesus (information from Lonie, 1981, p. 176) |
|  |  | Aristotle | Thompson, (1910) | Described in the chicken egg (‘a membrane’) but not named (Thompson, 1910, p. 561b n. 5); Thompson doubts the authenticity of this part of the text |
|  |  | Malpighi | Malpighi (1673) |  |
| angioblast | A tissue primordium (Gewebsanlage) that gives rise to both blood and capillaries | His | His (1900, pp. 325–326) | His’s term is still used today for endothelial precursors (cells rather than tissues; Risau & Flamme (1995, p. 78) |
| anterior intestinal portal | Opening of the foregut into the yolk sac or midgut | von Baer and Remak | Remak (1855, p. 12); von Baer (1837, p. 119) | von Baer’s Vorderer Eingang in den Darmkanal (Aditus anterior ad intestinum), Remak’s vordere Darmpforte |
| area pellucida, area vasculosa | Translucent (central) and vascular (peripheral) areas, respectively, of the early chicken embryo | Wolff | Wolff (1768) | Areola pellucida, area vasculosa, Figs. 1, 5, 6 |
| blood island | Cluster of haemopoietic and vasculogenetic cells in the blastoderm | Pander | Pander (1817) | Pander says (p. 14) that these were first described by Wolff (no citation given) |
| brain vesicles | Dilatations of the neural tube that give rise to the forebrain, midbrain and hindbrain. These primary vesicles were then subdivided into five categories | Malpighi | Malpighi (1673) | cerebri vesicule, at first three (p. 9, Figs 10–12); then five (p. 16, Fig. 18) |
|  |  | von Baer | von Baer (1837) | He named the five vesicles from rostral to caudal: Vorderhirn, Zwischenhirn, Mittelhirn, Hinterhirn, und Nachhirn (p. 107) [prosencephalon, diencephalon, mesencephalon, metencephalon, myelencephalon] |
| branchial clefts | (in amniote embryos) | Rathke | Rathke (1825) | Rathke describes lens-shaped openings in amniote embryos that connect the pharynx with the exterior, the first of which has a part that is similar to the operculum of fishes |
| buccopharyngeal membrane | the endoderm–ectoderm double membrane closing off the embryonic oral cavity from the exterior | Remak | Remak (1855, p. 79 n. 54) | Rachenhaut |
| chorda dorsalis | notochord | von Baer | von Baer (1828 p. 28) |  |
| chorion | the membranes enclosing the foetus | Antiphon |  | information from Lonie (1981, p. 176) |
| cleavage | (of the fertilised egg) | Prévost and Dumas | Prévost & Dumas (1824) | Not named but described in the frog (pp. 110–114); the embryo is divided up and comes to resemble a raspberry (p. 112). The concept was later elaborated by Rusconi and by von Baer (reviewed by Morgan, 1897, pp. 48–49) |
| convergence-extension | gastrulation movements | Vogt? | Vogt (1929) | Streckung und Konvergenz |
| dendrite |  | His | His (1889, p. 363) |  |
| embryo–foetus transition in humans |  | His | His (1882*a*, p. 44) | At the end of the second month |
| endothelium |  | His | His (1865*b*, p. 18) |  |
| epi-spinal spaces or sinuses (of His) | Spaces within the pia mater and between the pia and the brain | His | His (1865*c*; His, 1867*b*) | Possible artefacts (Woollam & Millen, 1954) |
| face as the rostral surface of the head |  | His | His (1880, p. 12) |  |
| facial reconstruction | The technique of predicting the facial appearance in life, by superimposing, on a skull, average tissue-thickness values | Welcker | Welcker (1883) |  |
| floor plate, roof plate | Of truncal neural tube | His | His (1886, p. 483) |  |
| germ cells | Of neural tube | His | His (1889, p. 314) | Keimzellen. His used this term earlier (His, 1868*b*, p. 200) to describe embryonic cells *sensu lato* |
| germ layers |  | Wolff |  | Formation of the gut in the chicken embryo from a ‘membrane’ (membrana) layer (Wolff, 1768, p. 448); Oken derived the gut (in mammals) from the yolk sack apparently unaware of Wolff’s papers (Oken & Kieser, 1806, p. 3; Wolff, 1812, p. 5) |
|  |  | Pander | Pander (1817) | Keimhautblätter (p. 22) |
| heterochrony | Change in developmental timing between ontogenies (in one species, or between species) | Haeckel | Haeckel (1874, p. 634) | Used earlier, in pathology, to describe a timing abnormality in tissue formation (Virchow, 1859, p. 58) |
| histogenesis | The development of differentiated tissues from precursor cells | Valentin, Wagner | Valentin (pp. 214–229 in Wagner, 1841) |  |
| holoblastic, meroblastic | Describe particular cleavage patterns | Remak | Remak (1855, p. 82) | In holoblastic animals, the cleavage planes during cytokinesis divide the entire ooplasm; in meroblastic animals only part of the ooplasm is cleaved |
| isthmus of hindbrain |  | His | His (1880, p. 23) | ‘Isthmus des Hinterhirns’; isthmus rhombencephali ≈ midbrain-hindbrain boundary |
| hypothalamus |  | Waldeyer and His | His (1893*a*, p. 159) | His introduced the name in print but it was suggested by Waldeyer |
| lateral plates | The two mesoderm layers peripheral to the intermediate mesoderm | Remak | Remak (1855, p. 10) | Seitenplatten. Remak (n. 7 on pp. 10, 11) notes that they were first described by Wolff and by Pander but under different names (Bauchplatten or laminae abdominales and Bauchfalten or plicae abdominales, respectively) |
| lymph sinus |  |  | His (1862, p. 436) | Research carried out with his friend Billroth |
| lymphatic system as a closed system |  | His | His (1863*b*) | Reviewed by Sabin (1913, pp. 4–6). Mall (1905, p. 144) refers to His’s paper as the best on the subject |
| mantel layer | A layer of grey matter formed of neuroblasts surrounding the central canal of the neural tube | His | His (1886, p. 482) |  |
| megaspheres | Spherical masses (40–60 µm) of yolk in the chicken egg | His | His (1877*a*, p. 145) | Megasphären |
| midgut |  | Wolff | Wolff (1768, p. 459) | intestinum medium |
| morphogenesis | the ‘generation of new form’ or ‘change in form’ |  |  | The first definition quoted is from Torrey (1967, p. 45) but the concept is much older. The second definition is from Wolpert (1994, p. 571) |
| nervi vasorum | Nerves forming a plexus in the tunica adventitia and tunica media of blood vessels | His | His (1863*a*) | Described, but not named as such |
| neural crest |  | Discovered by His, named by Marshall | His (1868*b*) | Named ‘Zwischenstrang’ by His and ‘neural ridge’ by Marshall (1878, p. 11). Marshall later stated that the two pairs of ridges constitute a single structure, the ‘neural crest’ (Marshall, 1879, p. 305 n. 2). His’s ‘Zwischenstrang’ is derived from his ‘Zwischenrinne’ (intermediate groove, p. 74), which demarcates the neural plate from the epidermal ectoderm, and the axial from the parietal parts of the embryo more generally. |
| neural folds |  | Reichert | Reichert (1840) | see **neural plate** (below) |
| neural plate |  | Remak, von Baer?, Reichert | Remak (1855, p. 8) | von Baer’s Ruckenplatten (von Baer, 1828, p.14); his primordium of the vertebral arches may actually be the primordium of the central nervous system, which comprises two primitive halves (neural folds) according to Reichert (1840, p. 13) |
| neural tube |  | Malpighi | Malpighi (1673) | spinalis medullae (spinal cord), but not clearly illustrated |
|  |  | von Baer | von Baer (1828) | Nervenröhre (p. 155) |
| neurite |  | His | His (1904, p. 26) |  |
| neuroblast |  | His | His (1889, p. 314) |  |
| neuroglia |  | Virchow |  | ‘… a kind of glue, Neuroglia’ (Virchow, 1856, p. 890 n. 3) |
| neuron |  | Waldeyer | Waldeyer (1891, p. 52) |  |
| neuronal growth to target |  | His | His (1886 pp. 509–510) | Weiss’s ‘contact guidance’ theory is based in part on His’s ideas (Weiss, 1955, p. 356 |
| neurula |  | Rauber | Rauber (1877) | He writes (p. 64 n. 1) that the term was first used in a different sense by G. Jäger (but provides no citation) |
| primitive folds |  | Pander | Pander (1817) | Primitivfalten (p. 9) – actually the neural folds? See Remak (1855, p. 9 n.5) |
| primitive streak | Axenplatte of Remak (1855, p. 7) | von Baer | von Baer (1828) | Primitivstreifen (p. 12) |
| re-segmentation hypothesis | Development of somites into the vertebral column such that there is a parasegmental relationship between the two | Remak | Remak (1855, p. 42) | neue Gliederung |
| septum spurium | A fold in the roof of the right atrium | His | His (1885, p. 162) |  |
| septum transversum | (of body cavity) | His | His (1880, p. 126) |  |
| somites |  | Malpighi | Malpighi (1673) | duplici vertebrarum ordine [two rows of vertebrae] (p. 5, Fig. 8) |
| spina vestibuli | An extension of mesenchyme from the dorsal mesocardium into the atrium | His | His (1885, pp. 149–152) | dorsal mesenchymal protrusion (Snarr *et al*., 2007). |
| spongioblast | Supposed glial cell precursors in the developing central nervous system. | His | His (1889, p. 314) | His states (p. 314, n. 1) that the term was first used by W. Müller in a different sense. |
| telencephalon | Rostral division of the prosencephalon | His | His (1893*b*, p. 179) |  |
| tuberculum impar | Median tongue primordium | His | His (1885, p. 60) |  |
| vitelline vessels |  | Aristotle | Thompson (1910) | Described but not named (p. 561b n. 1) |
|  |  | Wolff | Wolff (1812) | Dottervenen (p. 77) |
| yolk sac |  | Aristotle | Thompson (1910) | Observed in the chicken egg but not named (p. 564b n. 4) |

# Table S3. Opinions of earlier researchers on the origins of the peripheral ganglia and the nephric duct.

The disputed issues were: (*i*) whether the neural crest (intermediate cord) is the precursor of the nephric duct, or of the peripheral nervous system; (*ii*) whether the neural crest arises directly from the epiblast, or indirectly from it *via* the neural tube (*iii*) whether the spinal ganglia are always in connection with the neural tube *via* their dorsal roots, or whether they separate completely from the neural tube and then give rise to the connecting dorsal roots *via* sprouting. His was unsure but thought that option (*iii*) was likely (His, 1879, p. 477). See also Table S2 (neural crest).

| A. Peripheral ganglia | | |
| --- | --- | --- |
| Suggested origin | **Reference** | **Notes** |
| mesoderm | Remak (1855) |  |
| ectoderm | Hensen (1864*a*) |  |
| ectoderm | Hensen (1864*b*, p. 180) |  |
| ectoderm (provisionally) | His (1865*b*) | pp. 7–9. His states that an ectodermal origin is likely, but higher-quality histology is needed to settle the issue |
| ectoderm | His (1868*b*) | p. 169; His argues that the ectoderm gives rise to the Zwischenstrang (intermediate cord). As a provisional hypothesis, the intermediate cord contributes cells to the cranial and spinal ganglia, substantia gelatinosa, auditory vesicle and olfactory capsule. A pair of structures similar to the intermediate cord was observed by Marshall and named the ‘longitudinal ridges’ (Marshall, 1877, pp. 496, 500). Marshall derived the ridges not from the ectoderm but from the dorsum of the neural tube. He later considered the ridges to be a single, unpaired structure which he renamed the ‘neural crest’ [Marshall, 1879, p. 305 n. 2; discussed by Beard (1888) and Hall (2008)] |
| dorsal neural tube | Balfour (1875) | p. 135 |
| dorsal neural tube | Balfour (1876) | pp. 177, 189 |
| ectoderm | Hensen (1876) | p. 375 |
| dorsal neural tube | Balfour (1878) | a single proliferation of cells in the dorsal neural tube, representing the primordium of a pair of dorsal root ganglia; each ganglion primordium is therefore initially in continuity with the other, and with the neural tube, forming a single primordium (p. 156); he says this is consistent with the views of Hensen (1876) |
| epiblast beyond the limits of the neural plate | Beard (1888) |  |
| (B) Nephric duct | | |
| Suggested origin | **Reference** | **Notes** |
| mesoderm | Remak (1855) |  |
| mesoderm | Kölliker (1861) | p. 111 |
| ectoderm | His (1865*a*) | p. 161: on sections of chick embryos with an open neural plate, there is a deep infolding (Urnierenfalte) of the ectoderm lateral to the plate, which His states is the Anlage of the Wolffian duct (Plate XI, Figs B–D). In one figure, he also notes a possible anlage of the Müllerian duct (Plate XI, Fig. A) |
| ectoderm | His (1865*b*) | pp. 7, 11 |
| ectoderm | Hensen (1867) | On pp. 369–370 Hensen (1867) writes that he has found evidence in support of the view of His that the nephric duct arises from the ectoderm. Hensen (1903, pp. 31–32) maintains his position and (pp. 41–42) defends his view against the now-opposing view of his friend His (after His had changed his mind). Hensen sticks to an ectodermal origin in mammals. |
| mesoderm | His (1868*b*) | p. 119: His retracts his previous claims and states that the nephric duct arises on the lateral face of the somite. He also renames the Urnierenfalte as Zwischenrinne. p. 167: His withdraws his claim that the Müllerian duct, like the Wolffian duct, stems from the ectoderm |
| mesoderm | Dursy (1866) | Dursy criticises His for deriving the nephric duct from the ectoderm (pp. 57–59). In Dursy’s opinion, the duct arises in the mesoderm between the somite and the lateral plate (p. 58) |
| mesoderm | Waldeyer (1870) | Waldeyer derives the duct from the ‘Axenstrang’, itself ultimately derived from the epiblast by midline ingression |

| Table S4. Models used by His and others to explain and understand developmental processes. His (1894) describes in detail models that are simple enough to be carried out by a reader at home. Ernst Haeckel was contemptuous of these models, although many developmental biologists used models and analogies as heuristic devices. For example, Pander demonstrated blastodisc folding by spreading a handkerchief on his supinated hand and then flexing his fingers (von Baer & Oppenheimer, 1986, p. 142). Roux modelled cleavage by dripping oil into a wineglass containing diluted alcohol; the oil drops, representing the blastomeres, formed aggregates (Roux, 1896, pp. 7–9). Another of Roux’s models was a thought experiment: pieces of dough, with the yeast unequally distributed between them, would rise at different rates and reach different sizes. In this way, cryptic heterogeneities in the embryo might be transformed into ‘visible complexity’ (Roux, 1895*a*, pp. 9–10).  Rhumbler (1902) used corset stays – small elastic steel bars or Taillenstäbe – to build a model of the blastula. Stays of differing sizes were made into circles representing cells of differing sizes. All circles were then tied together with soft wire to form the blastula contour (Rhumbler, 1902, Table XXVI, Fig. 1). Rhumbler suggested (pp. 463–469) that the whole process of gastrulation can be explained by mechanical causes, the most important of which is the tendency of endoderm cells to undergo migration. Other factors include differential cell growth, which exerts a lateral pressure, and a decrease in blastocoel-fluid volume which sucks endoderm towards the blastocoele cavity. | | |
| --- | --- | --- |
| **His’s model** | **Context** | **Reference** |
| A circular metal disc, clamped round the margin, and heated in some areas with flames | A conceptual model where the disc represents the blastoderm, the individual flames represent growth zones, and the rate of growth is proportional to the intensity of the flame | His (1867*a*, p. 623 n.) |
| The wrinkling of a piece of moist blotting paper | Buckling of the blastoderm under the influence of differential growth | His (1867*a*, p. 623 n.); His (1868*b*, pp. 52–53) |
| A rubber tube with an incomplete longitudinal slit cut on one side, and its margins spread apart | To model the development of the sinus rhomboidalis of the rhombencephalon and lumbar neural tube | His (1867*a*, p. 628; 1868*b*, pp. 87, 133; 1873*a*, p. 331) |
| The imagined modelling of a lump of clay or wax | To picture the embryonic facial primordia | His (1868*b*, p. 138) |
| Folding a rubber tube longitudinally on itself | To model the formation of the optic evaginations | His (1868*b*, p. 150) |
| Postal envelope analogy | The axial structures in the early embryo grow faster than more lateral structures; the latter are therefore shorter in axial length than the midline structures and the four corners, where the limb buds will develop, are left as protruding but rounded corners (see also Fig. 8B, C in our main article). His modelled this phenomenon by folding sheets of paper with a stiff insert down the middle; folding the paper so that the edges are shorter than the insert. He also pictured this process using leather models with a string down the middle; he claimed that the leather can be coaxed into adopting the form of an embryo | His (1868*b*, p. 154; 1870*b*, p. 34; 1875, pp. 28–30)  Haeckel criticised this ‘comical’ Briefcouvert– Theorie (postal envelope theory); Haeckel (1875, p. 27; 1874, pp. 627–628) |
| Lead sheet and leather models for understanding morphogenesis of the blastoderm | His found these models very useful in understanding the early folding of the blastoderm. For the more detailed modelling of later development, he carried out modelling with Ziegler using wholemounts and sections in modelling clay and wax. For the Ziegler models see Hopwood (2002) | His (1868*b*, p. 182) |
| Folding of a piece of paper along its long axis | His felt this linear folding was a better model of how the chicken embryo pinched off from the yolk sac than von Baer’s circular purse–string model (von Baer, 1837, p. 45) | His (1868*b*, pp. 45–46) |
| Fabric trimmings left over when a garment has been cut from the cloth by a tailor | An analogy of the evolution of vestigial organs such as the thyroid gland and hypophysis (organs for which no physiological function was then known) and which represent ‘trimmings’ | His (1868*b*, p. 56). Haeckel criticised this Höllenlappen-Theorie [infernal-trimmings theory], because, in Haeckel’s characterisation, the tailor casts them into the furnace (Haeckel, 1875, pp. 26–27; Haeckel, 1874, pp. 627–628) |
| A broad rubber band cut open, laid flat on a table, then curved in the plane of the table top | To investigate tensile and compressive forces (along its outer and inner curvatures, respectively) | His (1868*b*, p. 70) |
| Two strips of paper loosely glued together to model the gradual separation of the upper and lower germ layers | Unequal pressure on the two–layered strip will cause the two pieces to tear away from one another in stages | His (1868*b*, p. 61) |
| Rubber tubes (see main text) | To demonstrate brain development | His (1873*a*, p. 331; 1875, pp. 95–99) |
| The subordinate role of parablastic compared to archiblastic tissues | While the nerves, muscles and glandular tissues of the archiblast have highly specialised, irreplaceable functions, the role of parablastic tissues is more mundane. One could imagine that their function as supporting and connective tissues could easily be substituted by something with similar physical properties (wood, leather, canvas, etc.) and that blood could be substituted by an aqueous solution of salts. | His (1875, p. 43) |
| A pinched rubber plate embedded in clay which fixes its most sharply bent part | A model of events around the development of the axial cord (Axenstrang) | His (1875, p. 62) |
| A piece of wax or clay, moulded into the form of an early embryonic head (his Fig. 77) | Pinched in at the four corners, this model will come to resemble the embryonic face as shown in his fig. 78 | His (1875, p. 90) |
| Folding a sheet of paper to model the ‘bending elasticity’ [Biegungselasticität] of the blastoderm (its elasticity in flexion) | His modelled the folds in the blastoderm by taking a flat sheet of paper and pushing the edges inwards: a fold is produced. In an additional demonstration, he used a piece of dry paper and wetted the centre of it: wrinkles form, whereas the dry edges of the paper remain flat. A sheet of inelastic material such as a wax or clay, does not show the same wrinkling under stress as observed for the paper. His concluded that elasticity is a necessary condition for folding. This contradicted the claim of Haeckel that the blastoderm is inelastic (Haeckel, 1872, pp. 471–472 n. 3) and His requested the evidence for Haeckel’s denial | His (1875, pp. 48–49) |
| One long edge of a leather strip forced into a curve in the plane of its flat surface; as a result, the leather is thrown into a repeating series of folds | His argues that similar forces might cause intersomitic clefts to develop (see Fig. 7D in our main article). | His (1875, pp. 64–65) |
| A circular ring of rubber tubing (e.g. a rubber band) pushed inwards at one point on the margin to form an elongated, two-layered strip | Models the process of concrescence at the germ ring | His (1877*b*, p. 109, n.) |
| Segments of a telescope gliding over one another | Similarities to how the pharyngeal arches are stacked over one another, with each partly concealed by the arch on the rostral side | His (1885, p. 28) |

# Table S5. Table of Contents of ‘*Our Bodily Form*’ (His, 1875) with chapter summaries.

The chapter summaries are from the Table of Contents. ‘*Our Bodily Form*’ is dated 1874 on its title page, and 1875 on the hard cover and Foreword. This discrepancy in dates was noted by Haeckel (1875, p. 13, n. 1) and perhaps suggests that His was in a hurry to publish (Richards, 2008, p. 284 n. 21). We assume that the correct date is 1875 because (1) His was still exchanging letters with Miescher about ‘*Our Bodily Form*’ in the winter of that year (His, 1897*b*, p. 18); (2) because he signs it January, 1875 in the front matter (His, 1875, p. VI); and (3) because the book was advertised in *Gegenwart* on March 27th, 1875 (Fig. 8A in our main text).

| **German original** | | **Our translation** | |
| --- | --- | --- | --- |
| Erster Brief | Die embryonale Körperform und ihre Entstehungsgeschichte | First Letter | The embryonic bodily form and the history of its origin |
| Zweiter Brief | Princip der organbildenden Keimbezirke, dorsale und ventrale Flächen der Embryonalanlage und deren Sonderung; vorderes und hinteres Körperende; allgemeine Topographie der Keimbezirke | Second Letter | Principle of the embryonic organ-forming regions, dorsal and ventral surfaces of the embryo primordium and their differentiation; cranial and caudal ends of the body; general topography of embryonic regions |
| Dritter Brief | Die Schichten der Embryonalanlage. Keimblattlehre. Parablastische und archiblastische Anlagen | Third Letter | The layers of the embryonic primordium. Germ layer theory. Parablastic and archiblastic primordia |
| Vierter Brief | Faltenbildung im Keim und deren Bedingungen | Fourth Letter | Formation of folds in the embryo and their conditions |
| Fünfter Brief | Mechanik der Blätterspaltung, Einfluss der Keimscheibenspannungen auf die Form der Zellen. Überschreitung der Elasticitäts– und der Festigkeitsgränzen, Bildung des Axenstrangs und der Urwirbel, Bildung von Näthen. | Fifth Letter | Mechanics of layer-splitting, influence of tensions in the germinal disc on the form of the cell. Exceeding of elasticity and stability limits, formation of the axial chord and somites, formation of sutures |
| Sechster Brief | Allgemeinheit des Faltungsprincipes bei der Bildung von Organanlagen. Bildung von Herz, Luftröhre, Lungen, Leber, Schilddrüse, Magen und Milz | Sixth Letter | Generality of the folding principle in the formation of organ primordia. Formation of the heart, trachea, lungs, liver, thyroid, stomach and spleen |
| Siebenter Brief | Die weiteren Folgen vom Princip ungleichen Wachsthums. Die Folgen der Abflachung des Körpers; Umbildung des Gesichtes | Seventh Letter | Wider consequences of the principle of unequal growth. Consequences of body flattening; reshaping of the face |
| Achter Brief | Das embryonale Gehirn. Formen einer sich biegenden elastischen Röhre. Ableitung der ersten Gehirnformen | Eighth Letter | The embryonic brain. Forms of a bended elastic tube. Derivation of the first brain morphologies |
| Neunter Brief | Bedeutung der Brückenkrümmung für die Entwicklung des Kleinhirns und der Medulla oblongata; Hemisphären des Grosshirns und deren Umbildung. Auftreten der weissen Substanz | Ninth Letter | Significance of the pontine flexure for the development of the cerebellum and medulla oblongata; cerebral hemispheres and their remodelling. Appearance of the white matter |
| Zehnter Brief | Das Wachsthumsgesetz; räumliches und zeitliches Wachsthumsgefälle und deren Bedeutung für die schliessliche Ausbildung des Körpers | Tenth Letter | The law of growth; spatial and temporal growth gradients and their significance for the final development of the body |
| Elfter Brief | Theorien der Zeugung, Extract- und Präformationstheorien, Theorien formbildender Kräfte | Eleventh Letter | Theories of reproduction, extract theory and preformation theory, theories of morphogenetic forces |
| Zwölfter Brief | Die Theorien der übertragenen Bewegung | Twelfth Letter | The theories of transmitted motion |
| Dreizehnter Brief | Vermittelung erblicher Uebertragung. Die Descendenzlehre und die Beziehungen der Morphologie zu derselben | Thirteenth Letter | Mediation of hereditary transmission. The theory of descent and the relations of morphology to it |
| Vierzehnter Brief | Die Erklärung organischer Körperform durch das Descendenzprincip, das „biogenetische Grundgesetz“ und seine Begründung. Unmittelbare und mittelbare Erklärung | Fourteenth Letter | Explanation of organic bodily form by the principle of descent, the ‘biogenetic law’ and its rationale. Direct and indirect explanation |
| Fünfzehnter Brief | Die Beziehungen embryonaler Formen zu einander; die erste Entwicklung des Amphioxus und des Petromyzon verglichen mit derjenigen von Knochenfischen | Fifteenth Letter | Interrelations of embryonic forms; first development of *Amphioxus* and *Petromyzon* compared with that of Osteichthyes |
| Sechszehnter Brief | Ueber die spezifische Physiognomie jüngerer Embryonen | Sixteenth Letter | On the specific physiognomy of younger embryos |
| Siebzehnter Brief | Beziehungen zwischen Descendenzprincip und Wachsthumsprincip. | Seventeenth Letter | Relations between the principle of descent and the principle of growth |
|  | Schlusswort |  | Closing words |
| Bemerkungen |  | Comments |  |

# Supplementary Note S1. Additional information on the oil painting of Wilhelm His shown in Fig. 1D of the main text.

The face, hair and clothing strongly resemble those in the formal portrait painted when His was appointed *Rektor* of Leipzig University in 1882 (Fig. 6A in Hopwood, 2012). Cornelia Jung of the Kustodie of the University of Leipzig tells us that the signature and date on this portrait painting are difficult to read because of darkening of the varnish. However, the signature appears to read ‘Albert Winther’, and the first three digits of the date are possibly 189, the fourth being illegible. An artist by that name is recorded in Vollmer (1980, p. 93). According to that source, Winther (1851–1935) was a Danish-born painter who settled in Leipzig and painted at least one other Leipzig University portrait. It is possible that the painting was commissioned to mark a special occasion in His’s Leipzig University career such as his appointment as professor (1872), dean of the Medical Faculty (1878, 1888, 1889) or as *Rektor* (1882). If the painting does indeed date to the 1890s, as the partial date on the canvas suggests, then His would have been 59 or older when the portrait was painted. This hardly seems likely in view of his appearance in the painting. If His was in his twenties when the painting was made, this would pre-date the birth of Albert Winther. We assume that this is a Leipzig-related commission from the 1870s or 1880s.

The label on the back of the canvas reads: “Wilhelm His / Portrait v. Winther. Leipz. / Geschenk v. Fr. Elisabeth His 1922 / Wilh. His aus Basel, geb. 9.7.1831/ gest. 1.5.1904/ Anat. Prof. 1872 – 1904/ Anat. Inst.– Leipzig”. We thank Cornelia Junge for this information.

_________________________________

# Supplementary Note S2. Contributions of Wilhelm His Sr. to anthropology and forensic craniofacial reconstruction.

His published several anthropological studies including an important monograph on Swiss skull types (His & Rütimeyer, 1864). He also published an early example of cranio-facial reconstruction using skeletal remains (His, 1895*a*; reviewed by Zegers *et al.*, 2009). His used a modification of Welcker’s technique (His, 1895*a*; Welcker, 1883) in which measurements are made of the *post mortem* thickness of the soft tissues at different points on the face (Welcker, 1883, p. 58, figure; p. 59, table).

His applied this technique to skeletal remains that had been disinterred at the Johanniskirche, Leipzig, and that were anecdotally those of Johan Sebastian Bach. His and his sculptor Seffner created a model using tissue-thickness measurements made by His on cadavers, and compared it with various contemporary portraits of Bach. They claimed a positive identification of the remains as those of Bach. Mall describes the Bach project as ‘inductive anatomy’ based on measurements. His, like Welcker before him, was trying to reconstruct the appearance of a historical celebrity (Vanezis & Vanezis, 2000). Today, such reconstructions would be regarded as questionable, even though the tissue-thickness data set that His compiled is of good quality (Stephan, 2015*a*).

One problem is that His’s reconstruction may not have made blind (Stephan, 2015*b*, p. 2; C.N. Stephan, personal communication). His and his sculptor both studied several portraits of Bach (His, 1895*a*), and used these images expressly to help them reconstruct Bach’s features (His, 1895*a*, p. 418). The sequence of events is not entirely clear but if they did indeed examine pictures before making the model, then one could argue that they used *a priori* knowledge to reach a desired result.

Further, it is at least possible that His had decided at the outset that the remains were those of Bach. On the second page of his paper, he already labels the Johanniskirche skull as ‘The Bach Skull’ (p. 382, figure legend) and he refers in the title of his paper and throughout the text to the ‘Bach’ remains. Without apparent irony, his student Mall says, approvingly: ‘lt was found that the reconstructed bust presented all of the characteristics of Bach even more pronouncedly than do his portraits.’ (Mall, 1905, p. 150). Mall said that this project gave His an interest in what His called the inductive anatomical method (Mall, 1905, pp. 150, 158).

_________________________________

# Supplementary Note S3. Contributions of Wilhelm His Sr. to developmental neurobiology.

Wilhelm His (1831–1904) towers over the field of research on histogenesis of the nervous system in the 19^th^ century. (Jacobson, 2005)

His made important contributions to human developmental neurobiology (reviewed by DeFelipe, 2015; Dupont, 2018; Fick, 1904; Glover *et al.*, 2018; Haymaker & Schiller, 1970; Jacobson, 2005; Rasmussen, 1970). His discovered the neural crest, naming it the ‘intermediate cord’ [*Zwischenstrang*; His, 1868*b*, pp. 45, 78, 105; Beard, 1888; Bronner & Simoes-Costa, 2016; Hall, 2008; Le Douarin & Kalcheim, 1999; Fig. 4 in our main text; Table S3].

As noted by Kölliker (1853), it was unclear whether nerve fibres ended independently or whether, according to the reticular theory, they anastomosed with one another to form a reticulum or network. It had long been suspected that nerve fibres are outgrowths of neuronal cell bodies (Bidder & Kupffer, 1857; Kölliker, 1853) although this was only one of several theories about their origins (reviewed by His, 1883, p. 164). His supported the Bidder–Kupffer theory, arguing moreover that the cell body was the genetic, nutritional and functional centre of the nerve fibre (His, 1886 p. 513). His was recognised as one of the originators of neuron theory, which postulates that all the elements of the nervous system are neurons together with the processes that grow out from them (reviewed by Haymaker & Schiller, 1970; Lopez-Munoz, Boya & Alamo, 2006; for a critical review of neuron theory, see Nissl, 1903). This and other work led to His being nominated twice for the Nobel Prize for medicine and physiology (Table S1).

His discovered the nerves (*nervi vasorum*) that innervate blood vessels (His, 1863*a*), and by injecting silver nitrate solution into brain and spinal cord preparations, he was able to identify what he called ‘perivascular lymphatic spaces’ in the central nervous system (His, 1865*c*,*b*). In His’s illustrations (His, 1865*c*, plate XI), the spaces are seen ensheathing the blood vessels that ramify in the tissue of the central nervous system (CNS), intervening between the tunica adventitia of those vessels and the nervous tissue of the white and grey matter. He described them as being continuous with spaces – the so-called ‘epi-spinal spaces of His’ (Woollam & Millen, 1954) – that lie within the pia mater and between the pia and the brain (His, 1865*c*, pp. 135–136).

His regarded this lymphatic system of the CNS as having what he called a ‘mechanical’ function, namely, helping to protect the nervous tissue from potentially injurious surges of blood or tissue pressure. He thought this was accomplished by the temporary dilation of the pial spaces (His, 1865*c*, pp. 138–139). The inadequacies of histological techniques at that time meant that the precise location and histological nature of the spaces was unclear; the spaces described by His may have been artefacts (Woollam & Millen, 1954).

His introduced the terms floor plate and roof plate (His, 1886 p. 483) mantel layer (His, 1886 p. 482), isthmus (of the hindbrain; His, 1880, p. 23), dendrite (His, 1889, p. 363), and neuroblast (His, 1889; see Table S2). He distinguished the foetus and embryo in human development (His, 1882*a*, p. 44). His proposed that nerve fibres grow from their origin into the periphery (or to targets in the CNS) using spaces in the tissues as pathways (His, 1886 pp. 509–510). He further suggested that the growing nerve fibres can be diverted by obstacles such as cartilage or blood vessels.

Weiss acknowledged that his own ‘contact guidance’ theory of neuronal growth was based in part on His’s ideas (Weiss, 1955, p. 356).

_________________________________

# Supplementary Note S4. The conflict between Wilhelm His and Ernst Haeckel.

By chance I have known your Leipzig biologists personally for a long time and so I can imagine the proud regret with which these “exact researchers” will look down on your speculative aberrations… and the craftsman His, whose enthusiasm was already matched by his narrow-mindedness when he was a student…. (Ernst-Haeckel Archiv, No. 33194: Ernst Haeckel to Johann Karl Friedrich Zöllner, Jena 30th January, 1873, p. 4; also cited by Rádl, 1909, p. 168)

[Haeckel] felt His a particular foe, as well he might have; and his polemics against him, since they were inadequate to combat His on his own grounds, descended to ridicule of the most inane sort. (Oppenheimer, 1955, p. 17)

The conflict began innocuously in 1868 when His stated that his ‘growth law’ provided a sufficient explanation for development, and that the evolutionary history of an organism was irrelevant. In 1870, His went on to criticise unnamed ‘young adherents’ to Darwinian theory for advocating the biogenetic law as a mechanistic explanation of development (His, 1870*b*, p. 30).

Haeckel’s response came in his monograph on calcareous sponges (Haeckel, 1872). He referred to His by name, writing that His’s attempt to give a mechanical explanation for development was misguided and ‘worthless’ (Haeckel, 1872, p. 472 n.). Haeckel continued the quarrel in his book ‘*Anthropogeny*’, a semi-popular survey of human evolution and development (Haeckel, 1874). In it, Haeckel casts doubt on His’s theories of development and on his scientific credentials, accusing him of lacking knowledge of comparative anatomy or phylogeny (Haeckel, 1874, pp. 52, 161, 627–629). The debate culminated in 1875 in heated polemics, when His published ‘*Our Bodily Form*’ (His, 1875) and Haeckel wrote the essay ‘Ziele und Wege …’ (Haeckel, 1875). A difference in opinion about science had developed into a feud [Nyhart, 1995, p. 340; see also Hopwood (1999) and Richards (2008)]. His and Haeckel both used the medium of the popular science book to express some of their harshest opinions, perhaps because they were then unconstrained by editors and peer-reviewers.

In principle, the apparently irreconcilable views of His and Haeckel are not mutually exclusive. For example, one could argue that the mechanical properties and growth rates of the embryo could evolve under natural selection — in which case there is merit to both sides of the argument. Rauber suggested this when he argued that His’s folding principle could lead to evolutionary changes in bodily form (Rauber, 1875, p. 258; Rauber, 1876, p. vi).

Regarding Haeckel’s polemics in general, Rádl (1909, p. 274) said that they: ‘… are coarse: no analysis, no discussion, no evidence, but only harshness …’. Considering the effect of His’s attacks on Haeckel, Rádl said: ‘Such derogatory judgments had little effect on [Haeckel]. … he had his listeners, his followers, and his theory spread despite all the protests (Rádl, 1909, p. 176). Braem (1895) noted that it was the very certainty with which Haeckel’s expounded his ideas that raised doubts about them. As for ‘*Our Bodily Form*’, one reviewer called it ‘very largely controversial’ and said: ‘it has always appeared to us a sign of weakness when a scientific combatant brings his quarrel before a general public’ (M.F., 1875).

_________________________________

# Supplementary Note S5. Other polemics and disputes.

Disputes can provide insights into what are perceived as particularly contentious scientific ideas, or into personal grievances regarding the feeling that one’s work has been plagiarized or inadequately cited.

We considered above the His–Haeckel dispute; His’s disputes with August Rauber are considered by Brauckmann (2006). Below, we consider His’s disagreements with Alexander Goette and Emil Dursy.

We do not think that His was especially conflict-prone; angry disagreements, played out in print, were not uncommon among his peers. For example, there was acrimonious conflict between Mehnert and Keibel regarding the extent of individual variation in the development of mammals compared with chelonians (Keibel, 1899; Mehnert, 1899; Gould, 1977, pp. 174–175; Hopwood, 2007, p. 13; Keibel, 1897, pp. 74–82). Rabl and Beard fought over the origin of the spinal ganglia, with Beard agreeing with His that ganglia developed directly from the epiblast but differing from His in viewing the Zwischenstrang as simply that leftover part of the epiblastic ingrowth that did not contribute to the ganglia (Beard, 1888, p. 161). In defence of his views, Beard wrote:

Professor [Oscar] Hertwig has thought fit to illustrate his account with one or two figures from as yet unpublished researches of Professor Rabl. If Professor Rabl is to pose as an authority on the formation of spinal ganglia, one may at least ask for tolerably correct figures in illustration of his work. The two figures 175 and 171 given by Hertwig are among the most incorrect that have been published till now on this matter.’ (Beard, 1888; p. 162; see Hertwig, 1888, fig. 171, p. 273 and fig. 175, p. 279)

The figures referred to by Beard, especially fig. 175, show the peripheral ganglia developing as an outgrowth from the dorsal neural tube. Rabl replies to Beard’s harsh words by inviting Beard to bring his histological preparations to the next scientific congress so that they could be compared side-by-side with his (Rabl, 1889, p. 224).

### (*a*) *His and Alexander Goette*

Goette, in his ‘*Development of the Fire-bellied Toad*’ (Goette, 1875, pp. 256–257) made a number of criticisms of His’s chicken embryo monograph (His, 1868*b*) and other works. Goette stated that while His was the only embryologist then to have attempted a mechanical explanation of development, his explanation only addressed the development of a few primordia when the embryo is already formed. For His’s folding mechanism, Goette suggested that many of the blastodermal folds described by His do not exist and probably represent artefacts. He criticised His for teleological arguments, where the development of a part is determined by its later function. He argued that His followed von Baer so closely that he repeated von Baer’s errors, including the antiquated concept of animal and vegetative layers, and von Baer’s mistaken concept of the primitive streak. Goette also suggested that His, in his microscopical observations, was guided by preconceived ideas.

His replied a year later, when he reviewed Goette’s monograph for the journal *Zeitschrift für Anatomie und Entwickelungsgeschichte* (His, 1876) of which he was a co-editor. In his review, His praised Goette for the quality of the Plates — but for little else. He acknowledged Goette’s work as a useful contribution to the struggle to preserve embryology as an independent discipline, free from the influence of dogmatic evolutionists. He criticised Goette for a lack of civility in his polemics against scientists who do not share his views. He also took issue with many points of Goette’s scientific argument and criticised him for not supporting his arguments with detailed measurements.

### (*b*) *His and Emil Dursy*

In ‘The Primitive Streak of the Chick’ Emil Dursy criticised His for deriving the nephric duct from the ectoderm (Dursy, 1866, pp. 57–59). In Dursy’s opinion, the duct arises in the mesoderm between the somite and the lateral plate (p. 58). Dursy also criticised Robert Remak, stating that Remak’s observations on early chicken development were flawed, and that this ultimately led him to distrust Remak (pp. 3–4). Dursy referred to Remak ironically – sarcastically, even – as ‘Master Remak’ (p. 4).

Dursy’s criticisms were not well-received by His (1868*b*, pp. 50–51 and p. 50 n. 4). Remak was one of His’s most important scientific mentors, and His held him in high regard. His later wrote in his memoirs: ‘I am grateful for Remak’s encouragement that in large part has determined my own life’s work.’ (His, 1903, p. 27). His stated that the tone of Dursy’s critique was inappropriate in a scientific work, especially when directed against a researcher of Remak’s merit (His, 1868*b*, p. 50 n. 4). His then criticized Dursy’s technique, arguing that it had led Dursy into erroneous conclusions (p. 51).

In a subsequent publication on head development Dursy (1869) responded to His’s comments. Dursy acknowledged that his critique of His had apparently caused resentment but stated that it was factual and well founded (p. vii). He also claimed that his criticism of Remak was justified, since Remak had made several errors (Dursy, 1869, p. 224). Further, he accused His (p. viii) of using, but failing to acknowledge, Dursy’s observations published in ‘The Primitive Streak of the Chick’.

His must have been concerned by this dispute, because he wrote a pamphlet entitled: ‘Records in the matter of accusations raised by Professor E. Dursy against W. His.’ (His, 1868*a*; published by F.C.W. Vogel, Leipzig).

His began the pamphlet (p. 1) with a message addressed to Dursy:

Dear Sir,

In the Foreword and Addendum to your recent publication on the development of the head, you make a series of accusations against me, essentially suggesting that in my ‘First Anlage of the Vertebrate Body’, I took results from your ‘Primitive Streak of the Chick’ and other works, and tried to dismiss them, partly by arbitrary distortion and partly by failing to mention their source, and that I then tried to appropriate those results as my own. These are serious charges against my integrity, and since you made the same charges in front of your embryology students in the previous summer semester, after my book came out, I feel that it is desirable to examine publicly the basis of your charges.

His enlisted the help of Victor Hensen to adjudicate the dispute. The pamphlet reports Hensen’s conclusions and His’s own responses to Dursy. Hensen concluded that most of Dursy’s accusations were unfounded. His did, in fact, cite Dursy’s works frequently, Hensen stated. He also rejected Dursy’s complaints of plagiarism, in particular Dursy’s claim that one of His’s illustrations (Plate XII, His, 1868*b*) was heavily influenced by Dursy’s work. Hensen disagreed, saying that His’s plate is quite different in conception from Dursy’s and differs in numerous details. The only valid claims made by Dursy – as His himself acknowledged – were that His failed to cite Dursy’s statements about the somitocoele, although Hensen felt that this was of little consequence. Second, His did fail to cite Dursy’s work on the hypophysis. In his defence, His pointed out that he received that publication too late to consider it, and he did not think it necessary to write a special addendum describing it.

_________________________________

# Supplementary Note S6. Scientific opinion on the parablast and concrescence theories.

## (*a*) *Parablast theory*

Rauber (1877), Kollman (1884) and Waldeyer (1883) agreed with His that that there was a special precursor for connective tissue and blood cells, but argued, *contra* His, that the embryo was wholly zygotic in origin: ‘Blood and connective tissue arise from the mesoderm, not from the white yolk; all tissues are derived from the zygote and the secondary germ [parablast] of His does not exist.’ (Rauber, 1877, p. 44). Kölliker suggested that the granules in the white yolk were possibly fat droplets, but not nuclei (Kölliker, 1876, p. 50). Goette accused His of using his study of embryos to confirm his preconceived ideas (Goette, 1874, pp. 192, 156; Goette, 1875, p. 555).

Haeckel was sceptical of the parablast theory observing that the gastrula of Amphioxus (*Branchiostoma* sp.) has two primary germ layers which give rise to all connective and vascular tissues; it has no tissue corresponding to His’s parablast (Haeckel, 1874, p. 629). The parablast theory was also difficult to reconcile with new ideas about gastrulation. Kölliker’s theory, the basis of the modern view, was that the mesoderm was a zygotic tissue derived from the primitive streak (Kölliker, 1880, pp. 22–23).

According to Fick, His was constrained by his parablast theory until the 1890s, when he eventually abandoned it, partly because his own findings contradicted it (Fick, 1904, p. 188; His, 1882*b*; reviewed by Hertwig, 1883, pp. 120–121). In 1900, His reflected on his ‘much maligned’ parablast theory and stated that it was no longer sustainable (His, 1900).

Decline in support for the parablast theory can be followed in textbook entries over the years. Looking back, Rabl argued that the parablast theory ‘was so obstructive to the progress of science for many years’ because it led scientists along the wrong path (Rabl, 1915, p. 24). Current opinion, based on cell-labelling experiments and the expression patterns of molecular markers, is that the entire embryo is derived from the epiblast in all amniotes (Stern, 2004; Lawson, Meneses & Pedersen, 1991).

## (*b*) *Concrescence theory*

Like the parablast theory, concrescence was influential but controversial (reviewed by Kopsch, 1904; Morgan, 1895; Rabl, 1897). Its leading supporters and critics are listed by (Kopsch, 1904, pp. 15–16). Rabl was sceptical about concrescence but described it as ‘ … one of the most important developmental theories about the structure of the vertebrate body…’ (Rabl, 1897, p. xii). Kopsch, also a sceptic, nonetheless argued that: ‘the epoch-making significance of His's concept consists in the extraordinarily simple way in which the embryonic development of vertebrates and invertebrates can now be explained by the same processes’ (Kopsch, 1904, p. 4).

Ablation experiments refuted the concrescence theory (Katschenko, 1888; pp. 456–457; Morgan, 1895) as did vital labelling in urodele embryos (Goodale, 1911, p. 241; Smith, 1914, p. 260). The decline in its influence can be tracked in the textbooks of the time. Today, concrescence has been replaced by models such as convergence-extension (convergent extension; Kunz, 2004, pp. 209–210; Keller *et al.*, 1991; Keller, Shook & Skoglund, 2008; Shindo, 2018).

_________________________________

# Supplementary Note S7. His’s ‘mechanical’ developmental biology: its meaning and its reception.

Since the mechanistic or mechanical approach of Wilhelm His can be misunderstood, we consider here some the different meanings of these terms as they have been applied to His’s work. See also (Oxford Dictionaries, 2017; Woodger, 1929, pp. 229–272; Driesch, 1891, pp. 2–6). The relevant meanings are:

(*i*) physical forces and the laws of Newtonian mechanics, as illustrated by the following quotations regarding the reductionist approach of His and Ludwig:

One of the earliest attempts to give a quasi-mechanical explanation of organ formation was that of Wilhelm His ('74). In a remarkable book entitled "Unsere Körperform" he approached the study of embryology from a very different point of view from that in vogue at the time. This book is one of the earliest attempts to escape from the historical treatment of the problem of development – a method that was in full swing in His' time under the guidance of Haeckel and other popular writers. (Morgan, 1927, pp. 225–226)

… [Carl Ludwig] sought to explain vital phenomena in terms of mechanics, that is, through laws of physics and chemistry. (Rosen, 1973, p. 540)

His taught his readers to see the formation of the chick embryo mechanically, inviting them to imagine that they were bending and folding layers of embryonic tissue. (Hopwood, 1999, p. 470)

This mechanistic theory [of His] was greatly influenced by Carl Ludwig, professor of physiology at Leipzig, to whom His dedicated *Unsere Körperform* (1874), as a kind of mechanistic embryological manifesto. (Jacobson, 2005, p. 373)

(*ii*) causal mechanisms of development:

On the other hand, Wilhelm His, having overthrown Haeckel's theory of recapitulation, regarded each stage of development as a sufficient cause of the following stage, and so paved the way for a new branch of science: Entwicklungsmechanik or causal embryology, the foundations of which were laid by Wilhelm Roux. (Huxley & de Beer, 1963, p. 9)

(*iii*) materialism as opposed to vitalism:

Mechanistic materialism of the kind upheld by His, Moleschott, Ludwig, and others was unable to refute vitalist theories because there always remained some phenomena which could not be given a completely mechanistic explanation. (Jacobson, 2005, p. 373)

Or a mixture of these meanings:

First, it should be emphasized here that the "vitalistic" and "teleological" judgments of organisms, as we have already done, are assumed to be identical, and that the "mechanical" method, which in turn coincides with the "causalist", can be contrasted. …. Thus, this dogma of the vital force or the final causes stands in a sharp and irreconcilable antagonisms to the "mechanical" or "causal" conception, according to which life is a manifestation of motion, which differs only by its more complex composition from the simpler physico-chemical "forces" of the anorgans (minerals, water, atmosphere), and which just as inseparably connected with the more complex matter of the organism as the physical and chemical properties of the anorgans with their material substratum. (Haeckel, 1866*a*, p. 97)

Many scientists criticised His’s mechanical approach because it tried to infer developmental mechanisms from descriptive data. Despite this criticism, His’s research was important because it stimulated the study of developmental mechanisms, and because it provided a challenge to phylogenetic embryology.

Holtfreter (1943) described ‘*Our Bodily Form*’ as a pioneering work on the mechanisms of morphogenesis, but argued that His’s theories were unproven. Holtfreter pointed out that cell division during early amphibian development leads not to growth, as predicted by His’s theories, but to the production of smaller blastomeres. Similar conclusions were reached for killifish by Kessel (1960).

Mehnert suggested that His’s great merit was in his emphasis of the mechanical basis of embryological processes. He characterised His as a pioneer of developmental mechanics, along with von Baer (Mehnert, 1898, p. 76). Thompson (1917) credits His for emphasising the embryological aspects of differential growth (p. 55) and for his attempts ‘to import into embryology, wherever possible, the simpler concepts of physics, [and] to introduce along with them the method of experiment …’ (p. 57).

Hertwig described His’s attempts to model development in terms of the stretching of elastic plates as unrealistic (Hertwig, 1892, pp. 155–156). Keibel noted that His’s mechanical-developmental approach was of paramount importance, but that it lacked the necessary mathematical underpinnings (Keibel, 1906, p. 165). In Rabl’s view, His was right to argue that development and growth should be explicable in mechanical terms and he considered His to be one of the founders of developmental physiology [Entwicklungsmechanik] (Rabl, 1909*b*, p. 97). Similar sentiments were expressed by Wilson (1895, p. 118).

_________________________________

# Supplementary Note S8. Mosaic *versus* regulatory development.

Many experiments have been carried out to test the competing hypotheses of mosaic *versus* regulatory development. Wilson (1904) cut the unfertilized eggs of marine molluscs (*Dentalium* sp.) into fragments and then fertilised them. The incomplete eggs developed into embryos lacking specific structures, suggesting that the unfertilised egg was a functional mosaic. Wilson (1904) concluded (p. 70) that ‘The development of the molluscan egg is in its essential features a mosaic-work and sustains the theory of "Organbildende Keimbezirke." [organ-forming regions of His]’.

By contrast, Driesch (1892) separated the blastomeres of two-cell stage echinoderm embryos (*Echinus microtuberculatus*) by shaking them vigorously. The result was a morphologically complete, but half-sized embryo; Driesch concluded that the result refutes the principle of organ-forming regions (p. 178). Goette also disagreed with the concept of regionalisation: he considered the egg to be homogeneous (Goette, 1875).

Wilhelm Roux found the concept of organ-forming regions interesting and cited it frequently (e.g. Roux, 1895*a*). However, Roux argued that it was a descriptive concept, rather than a causal or functional one (Roux, 1895*a*, p. 825). Roux killed one of the blastomeres of a two-celled embryo of *Pelophylax esculentus* [*Rana esculenta*] with a hot needle (Roux, 1895*a*, pp. 419–521). Several such embryos developed into morphologically incomplete ‘half-embryos’ of various types. Roux interpreted his findings as supporting the mosaic theory (Čapek & Müller, 2019, pp. 1–2).

To reconcile the conflicting data, Wilson suggested that early embryos are indeed mosaic in all species, but that mosaicism begins at different stages (Wilson, 1893, p. 615). This hypothesis is similar to the concept of a totipotency–pluripotency transition (Boiani *et al.*, 2019). Amniote embryos are now generally thought to show regulative development – contrary to His’s view of organ primordia already mapped out in the egg – but the details of the regulative–mosaic transition are not completely known (Stern, 2006).

_________________________________

# SUPPLEMENTARY REFERENCES

All references cited in the Supporting Information are listed in the reference list of the main article.
